# Should we adopt the case report format to report challenges in complicated evidence synthesis? A proposal and illustration of a case report of a complex search strategy for humanitarian interventions

**DOI:** 10.1002/cesm.70021

**Published:** 2025-04-13

**Authors:** Chris Cooper, Zahra Premji, Cem Yavuz, Mark Engelbert

**Affiliations:** ^1^ University of Bristol Medical School, Bristol University Bristol UK; ^2^ Libraries, University of Victoria Victoria British Columbia Canada; ^3^ International Initiative for Impact Evaluation (3ie) New Delhi India; ^4^ International Initiative for Impact Evaluation (3ie) and University of East Anglia Norwich UK

**Keywords:** EGM, evidence gap map, humanitarian, study identification, systematic review

## Abstract

Case reports represent a form of evidence in medicine which detail an unusual or novel clinical case in a short, published report, disseminated for the attention of clinical staff. This form of report is not common outside of clinical practice. We question if the adoption of the ‘case report’ might also be useful in evidence synthesis. This where the case represents a challenge in undertaking evidence synthesis and the report details not only the resolution but also shows the working to resolve the challenge. Our rationale is that methodological responses to problems arising in complicated evidence synthesis often go unreported. The risk is that lessons learned in developing evidence synthesis are lost if not recorded. This represents a form of research waste. We suggest that the adoption of the case report format might represent the opportunity to highlight not only a challenge (the case) but a worked example of a possible solution (the report). These case reports would represent a resting place for the case, with notes left behind for future researchers to follow. We provide an example of a case report: a complicated search strategy developed to inform an evidence gap map on the effects of interventions in humanitarian settings on food security outcomes in low and middle‐income countries and specific high‐income countries. Our report details the solution that we developed (the search strategy). We also illustrate how we conceptualised the search, and the approaches that we tested but rejected, and the ideas that we pursued.

## 1 INTRODUCTION

Case reports represent a form of evidence in medicine which detail an unusual or novel clinical case in a short, published report, disseminated for the attention of busy clinical staff [1, 2, 3]. This form of brief report is not common outside of clinical practice.

We suggest adopting the ‘case report’ format to document specific challenges in conducting evidence synthesis. This where the ‘case’ represents an original challenge ‐ one which arises within the broad scope of undertaking complicated evidence synthesis ‐ and the ‘report’ details the researchers attempts to resolve ‐ and their current solution to ‐ the case.

We anticipate that these case reports would have broadly the same aim(s) as in clinical practice: to briefly update evidence synthesis practitioners and evidence users about solutions to new challenges. The merits of clinical case reports are well‐described, but their ability to provide in‐depth understanding of phenomena, and to act as a mode of communication between practitioners and researchers, are merits that are particularly relevant for evidence synthesis practitioners [3].

We would, however, like to see one subtle difference. In reporting the solution, we propose that the ideas considered or tested which did not work, or were not chosen, even if reported only in note form, are provided alongside the solution. This would be a difference between a case report in a clinical setting and what we propose. To this end, it would be important that the case reports be published separately to the synthesis, so they have a word count specific to the case in hand, and so the report can be written concisely by researchers for researchers in accessible but technical detail.

In suggesting the idea for case reports in an evidence synthesis setting, our rationale is that methodological responses to complicated problems often go unreported. In a systematic review, you will read a report of what was done but seldom do you read about the challenges encountered on the way to a reliable synthesis. For instance, you will read the final draft of the search strategy, final conceptualisation of the inclusion criteria, or see a completed network plot for a network meta‐analysis. But you cannot get from this alone what challenges the authors faced and how they dealt with them. The risk is that lessons learned in developing evidence synthesis—or even errors made initially and corrected subsequently—are lost if not recorded. This represents a form of research waste [4].

So, our proposal is to detail the case and how it was solved, while also setting out the process of developing the solution, including any unsuccessful avenues explored. These case reports might then represent either one possible solution, or a resting place for a problem but, by detailing their workings, the hope would be that path to resolution is documented for future researchers to consider (even if they then go in another direction).

In proposing this idea, we have questioned ourselves what constitutes a good case: that is, what is a complex problem that should be documented in this way. Unsatisfactorily, perhaps inevitably, we think this: you'll know it when you see it (it probably will raise this discussion).

We can't go over it.

We can't go under it.

Oh no!

We've got to go through it [5]!

**Paper aim and structure**

We aim to illustrate our proposal for the adoption and use of case reports to report challenges arising in complicated evidence synthesis. The structure that we set out below is what we propose generally if this ‘style’ of report were adopted (namely: 1. The Case (description of the case). 2. The Response (what was done and why) and 3. Conclusions (or next steps).

**1. The Case**

**Figure 1:**
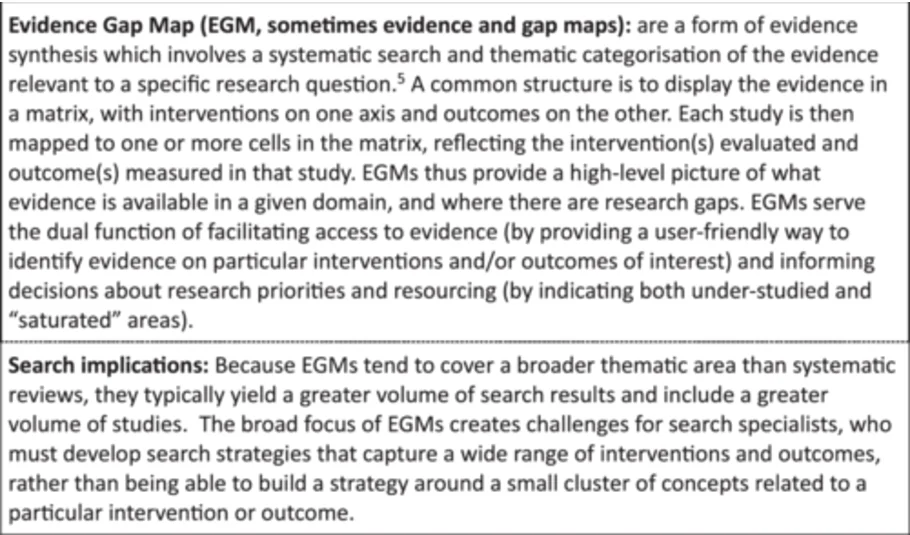
What are evidence and gap maps.

The task in this case was to develop a systematic search strategy for an EGM comprising impact evaluations and systematic reviews that describe evidence on the effects of interventions in humanitarian settings on food security outcomes in low‐ and middle‐income countries. EGM are defined in Figure 1.

The challenge we faced was capturing the EGM's rather abstract concepts in a clear and systematic search. We needed, therefore, not only to conceptualise and define humanitarian settings (to delineate the scope of the search) but then frame it in a way that captured the available evidence without overwhelming the research team. We were conscious that any limitations of our framing of humanitarian settings could be viewed as a defect in our search approach, and also impact the success of study identification [6].

The rationale for making this a case report is to record the work conducted in the search creation phase for future researchers tackling similar challenges. We—and others—have noted elsewhere that reporting the work in the creation and development phase of a search strategy is often omitted with only the final draft of a search strategy presented [7, 8]. The amount of work which goes into generating searches for mapping reviews, or reviews with a similarly broad focus, is significant, with the burden of the work falling upon the searcher [6]. As we shall detail below, this process involves considering and perhaps testing multiple approaches to identity the optimal balance of sensitivity and specificity such that the relevant literature is captured but the research team is not overwhelmed.

Table 1 reports the Population, Intervention, Comparator, Outcomes, and Study Designs (PICOS) for our review [9]. These were framed by 3ie and defined the scope of work. In our review, two of the search concepts, namely Low and Middle Income Countries (L& MICs)/specific High Income Countries (HIC) and the study designs (impact evaluations and systematic reviews), had established search filters which allowed us to focus on the remaining search concepts.

**Table 1:** The population, intervention, comparator, outcome(s) and study design for the EGM.

Criteria	Description	Possible search concepts
Population	Studies implemented in a humanitarian emergency consisting of participants residing in low‐ and middle‐income countries. We also included studies consisting of participants residing in a selection of high‐income countries, so long as the intervention was implemented in a refugee camp.	Humanitarian settings
		LMICs (3ie search filter available) + select HICs
Intervention	We included the following seven intervention categories: early warning systems; interventions pre‐arranging household finance for disasters; food, cash and other in‐kind transfers; agriculture and livestock interventions; nutrition interventions; market‐based recovery interventions; water security interventions.	Intervention group
Comparison	A study must have included a comparison group, though there were no exclusion criteria based on the comparison condition of a control group.	None
Outcomes	We included the following types of outcomes: agricultural production outcomes; food trade; markets; food decisions; hunger; economic; food safety; nutrition; food intake; sustainability of food security; health; composite measures of food security.	Food/food security outcomes
Study designs	We included both impact evaluations and systematic reviews. For impact evaluations, we included studies using an experimental or quasi‐experimental design. Systematic reviews which synthesized the effects of an intervention on outcomes were included.	Impact evaluations OR Systematic reviews (3ie search filter available) combined

## 2 THE RESPONSE

### 2.1 Search creation phase (developing strategy structure)

We developed a test set of potentially eligible marker papers (*n* = 21). These were identified by CC through informal scoping of bibliographic databases, reference checking, and Google websearches. A second test set (*n* = 27) of unpublished reports, studies, evaluations, and types of evidence synthesis were provided by 3ie. The papers were screened to the inclusion criteria of the EGM by CY and determined eligible for inclusion. We merged both test sets making *n* = 21 articles in total (after removing duplicates), to form a set of marker papers for formally developing and testing the search strategy. We selected the sample of marker papers to ensure that key aspects of the PICOS were represented in the test set (see Table 1. e.g., some papers focused on the P, some on the I, and so on).

Secondly, we developed potential combinations of the PICOS structure and drafted search syntax to capture these. As the project was for 3ie, we used in all combinations a set of pre‐developed search filters for i) impact evaluations and systematic reviews, and ii) L& MICs/HIC. These filters are both used as standard in all 3ie projects and reviews and are reported here as part of the final search strategies. The study design filters have been developed iteratively over the past decade or so through the efforts of several information specialists who have introduced incremental improvements, often after encountering relevant studies that were filtered out by earlier versions of these filters. The L& MIC filters are those developed by the Cochrane group for Effective Practice and Organisation of Care (EPOC) [10]. While neither set of filters has been formally validated, they have proved effective as the basis for 3ie searches that aim to be highly sensitive while still keeping the number of search results to a level that is manageable given available resources on the typical 3ie project.

There were four trial approaches (see Table 2). The different combinations reflect different ways to frame the search around different aspects of the research questions. In particular, the approaches differ in whether/how they include food security outcome terms:

- Approach 1 focused on capturing the “humanitarian context” aspect within the research question. The terms developed to capture this concept were tested to assess retrieval of markers with the food outcome concept in two different ways (1a. focused vs. 1b. broad);
- Approach 2 focused on the types of intervention that were in scope, without restricting the results by conjoining (with “AND”) these terms with additional concepts for humanitarian contexts or food outcomes. The retrieval estimates were significant (as expected, *n* = 200,000 + ) so this approach was not ultimately considered viable. BUT, the terms developed for this approach were retained in Approach 3;
- Approach 3 used terms for humanitarian OR interventions, which were then conjoined with two versions of the outcome terms (as with Approach 1, 3a. focused vs. 3b. broad).
- Approach 4, focused on the outcome terms, namely food security. The proposal here was to follow an approach commonly used in searching for complex interventions in public health reviews. That is, where a question looks at ‘what is the effect of interventions to do X’ and the scope of interventions is not only unspecified but also numerous, and interventions may be poorly defined. By not specifying the interventions, but focusing on outcomes (or in some cases, on settings), the idea is to allow the screening phase to surface the full range of relevant interventions, rather than attempting to define these in the search strategy [11].

**Table 2:** Initial DRAFT search structures for testing against potentially eligible records.

Summary of approach	Estimated # of records to screen	Marker papers identified	Notes & recommendations
#1a humanitarian focused AND broad food 1. humanitarian concept 2. BROAD food/food security concept 3. 3ie filter for LMIC/refugee 4. 3ie filter for IR or SR 5. 1 AND 2 AND 3 AND 4	10–12K V. draft search + the 10 missed papers in doc below See Supplementary 1a	11 of the 21 marker papers	The humanitarian search (line 2) is good, but it does not deal with the specific mechanism of the interventions. It will identify the crisis or disaster angle (i.e., the setting) of any work but not the intervention or where food is not specified in title or abstract. **Suggest reject on this basis and IVO of similar retrieval performance of 1b.**
#1b humanitarian focused AND focused food 1. humanitarian concept 2. FOCUSED food/food security concept (e.g., (food etc adj3 (terms))) 3. 3ie filter for LMIC/refugee 4. 3ie filter for IR or SR 5. 1 AND 2 AND 3 AND 4	10–12 K The focused food search is under development. I include the five papers currently blocked by the humanitarian cluster in the embedded word file. See Supplementary 1b	13 of the 21 marker papers Three papers are blocked by the LMIC filter (the same three as for #1a) Five are blocked by the humanitarian cluster BUT WOULD be picked up the broad food OR methods cluster.	This is developed using the humanitarian cluster with a focused food security cluster. Question: are the five papers blocked by humanitarian cluster eligible (do I need to tweak the filter? This might be a valid approach, especially when combined with detailed supplementary searching, + with favourable numbers **Suggest combine this approach with approach 4.**
#2 humanitarian OR intervention 1. humanitarian concept 2. search concept for the classes of interventions 3. 1 OR 2 4. 3ie filter for LMIC/refugee 5. 3ie filter for IR or SR 6. 3 AND 4 AND 5	200,000 K (not a typo)	Not tested	Obviously insane numbers, and not a possibility, but I tested it and list for completeness. **Rejected for reasons stated.**
#3a humanitarian AND intervention AND broad food 1. humanitarian concept 2 search concept for the classes of interventions 3. BROAD food/food security concept 4. 3ie filter for LMIC/refugee 5. 3ie filter for IR or SR6. 1 AND 2 AND 3 AND 4 AND 5 AND 6	10–12 K See Supplementary 3a	11 of the 21 marker papers Two papers missed by the broad food cluster. Five papers missed by the humanitarian cluster. These seven papers would be picked up by all other clusters. I don't consider this a limitation of the humanitarian cluster, per se, rather an illustration of the practicable issue matching author reporting and inclusion criteria.	**Rejected as lower number of eligible studies identified. This cluster also performed less well in piloting.** The same three papers are missed by the LMIC cluster as at #1a.
#3b (humanitarian OR intervention) AND focused food 1. humanitarian concept 2. search concept for the class of interventions 3. 1 OR 2 4. FOCUSED food/food security concept (e.g., (food etc adj3 (terms))) 5. 3ie filter for LMIC/refugee 6. 3ie filter for IR or SR 7. 3 AND 4 AND 5 AND 6	60k+ This could be managed by tightening the methods cluster down, probably getting into the 20‐30k to screen range. See Supplementary 3b	18 of the 21 marker papers	The three which are missed are blocked by the LMIC cluster. **Rejected as n exceeds resource limits, but the core terms in the filters to be checked closely against 1b.**
#3c (humanitarian OR intervention) AND broad food 1. humanitarian concept 2. search concept for the class of interventions 3. 1 OR 2 4. BROAD food/food security concept 5. 3ie filter for LMIC/refugee 6. 3ie filter for IR or SR 7. 3 AND 4 AND 5 AND 6	45 K+ This could be managed by tightening the methods cluster down. See Supplementary 3c	16 of the 21 marker papers Two were blocked by the broad food cluster.	The same three were blocked by the LMIC cluster. **Rejected as *n* exceeds resource limits, but the core terms in the filters to be checked closely against 1b.**
#4 Food Focused 1. FOCUSED food/food security concept (e.g., (food etc adj3 (terms))) 2. 3ie filter for LMIC/refugee 3. 3ie filter for IR or SR 4. 1 AND 2 AND 3	45 K+ See Supplementary 4	18 of 21 marker papers identified	The three which are missed are blocked by the LMIC cluster. **Suggest to combine this approach with 1b as highest number of markers returned.**

We tested the marker papers in the Global Health database (Ovid Interface) against these combinations to evaluate which combination of approaches identified the maximum number of marker papers, and we examined why marker papers were missed by the draft syntax structures. We also considered the yield (namely, the number of items returned by each combination) and Number Needed to Read (the number of items needed to read until an eligible marker were identified) (see Table 3) [12]. Two combinations showed the most promise: 1b and 4. These approaches aligned most closely with the eligible markers papers. Approach 4 identified the highest number of marker papers overall, but had a high overall yield given that it used fewer concepts overall and did not include the humanitarian concept. Approach 1b had lower sensitivity (13 of 21 marker papers) but the loss was entirely attributed to the draft humanitarian concept (i.e., the search was run with and without the humanitarian concepts illustrating that when the humanitarian concepts were operationalised the search was less sensitive). Therefore, we decided to further refine the humanitarian concept to boost the sensitivity of Approach 1b to approach that of Approach 4 (which retrieved 18 marker papers) without resulting in the higher screening burden brought about by using one fewer search concept.

**Table 3:** Data from comparisons presented in Table 2.

	Final	1a	1b	2	3a	3b	3c	4
Yield (MEDLINE only)	6172	4090	4537	200,000+	4167	22481	20994	22718
Comparison to the 21 marker papers	18 (61.9%)	11 (52.3%)	13 (61.9%)	Not tested	11 (52.3%)	18 (85.7%)	16 (76.1%)	18 (85.7%)
Number needed to read	342	371	349		378	1248	1312	1262

### 2.2 Developing the humanitarian cluster

#### 2.2.1 Identifying other reviews and searches which have focused on humanitarian topics

Starting with the term "humanitarian," [13] we initially searched the Cochrane Library and Campbell systematic reviews, as these reviews often include detailed search strategies, even if they may not always meet the eligibility criteria for this EGM. Our objective was to identify any protocols or reviews evaluating interventions in humanitarian settings and to conduct an informal analysis of their search strategies, examining both the sources and methods used. We then expanded this search to other relevant databases, such as IBSS, LILACS, GEOBASE, and ASSIA, to identify additional systematic reviews.

#### 2.2.2 Websearching to identify definitions of ‘humanitarian’

We sought to identify an agreed definition of ‘humanitarian’, to ground this aspect of the search in established usage by scholars and practitioners in the field. Websites reported a distinction between man‐made and natural types of humanitarian disasters [14, 15]. We adopted this categorisation, using it to structure the search strategy. Figure 2 shows this structure with the ‘types’ highlighted in green, to illustrate the groupings.

Figure 2Humanitarian search cluster (including general, natural and man‐made crises terms). Formulated for the Ovid interface (MEDLINE database example).

**Figure:**
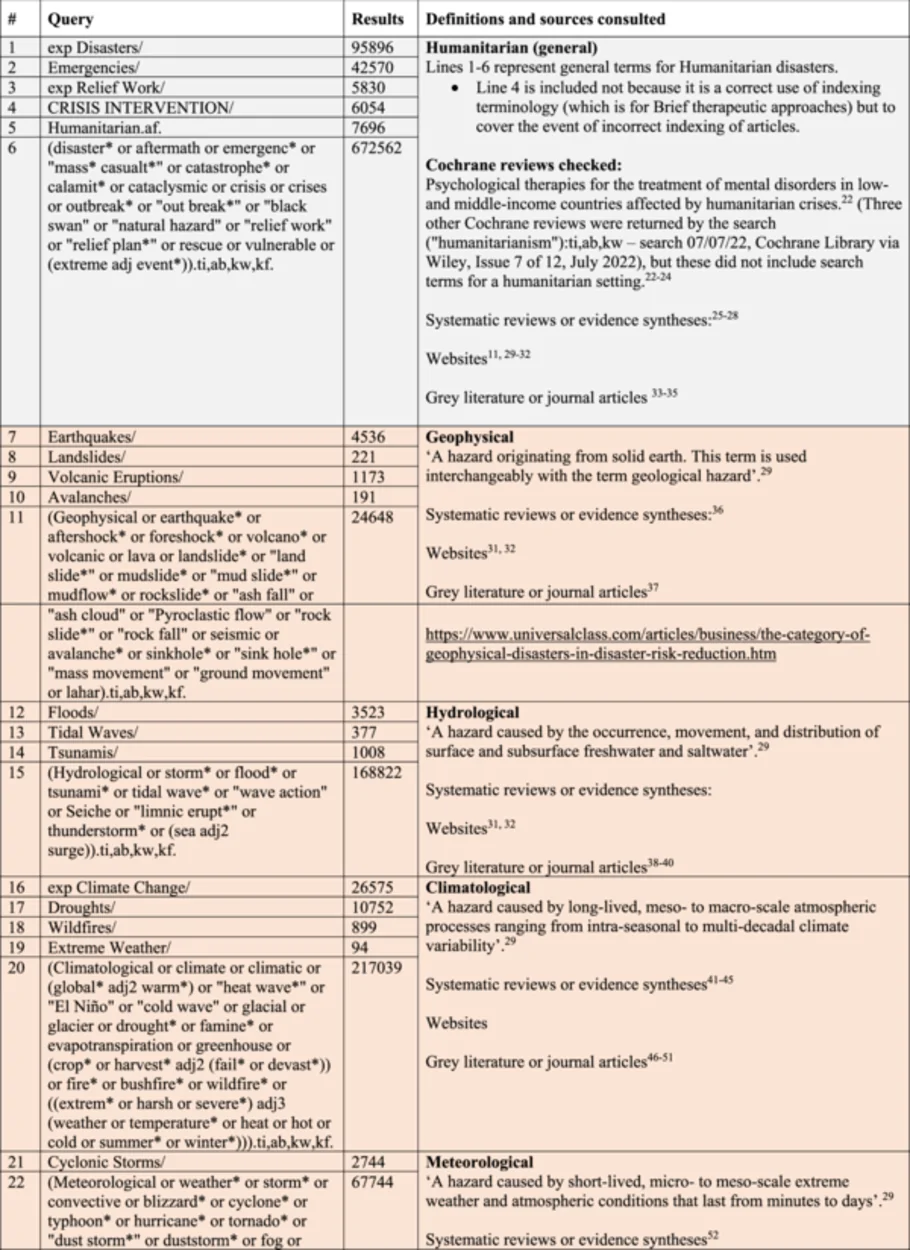

**Figure:**
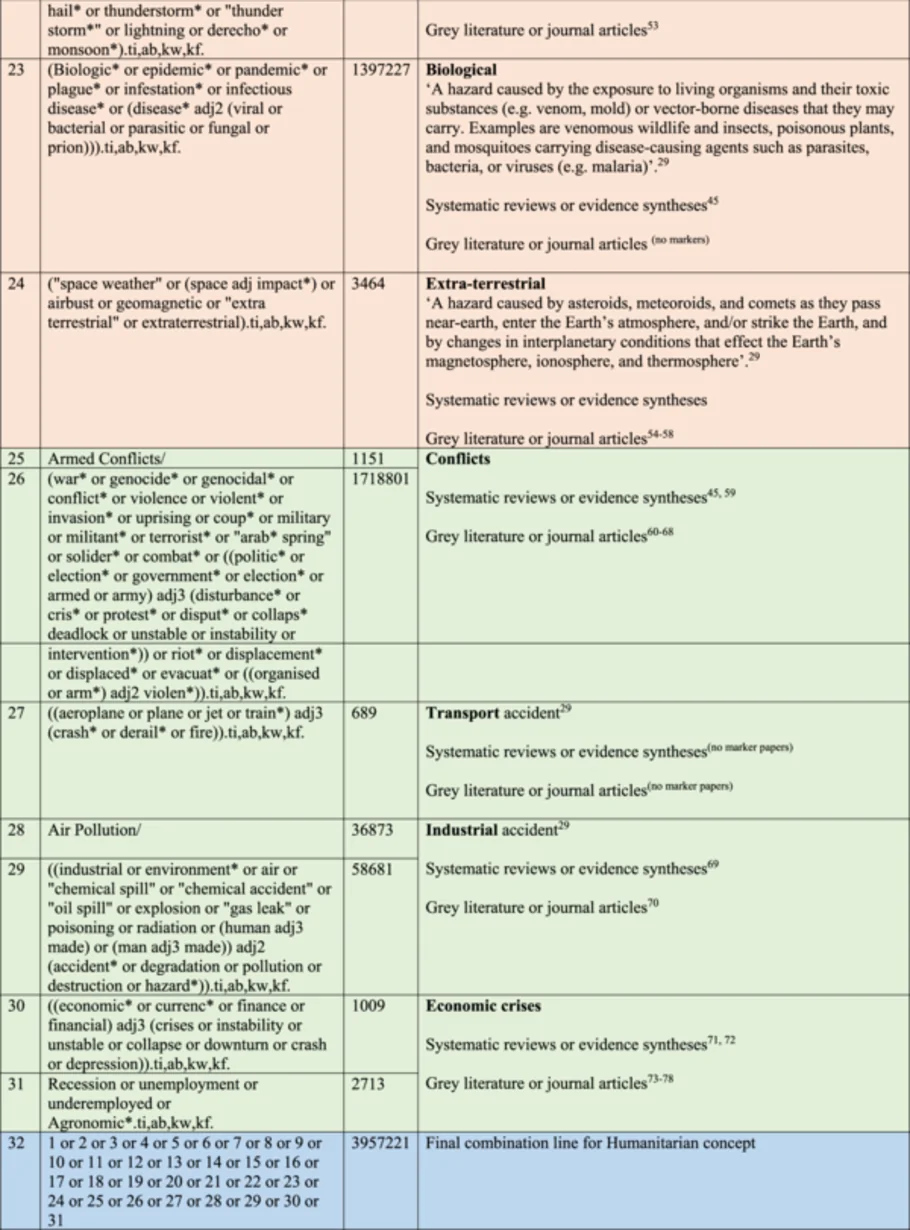

#### 2.2.3 Synthesizing definitions and developing a search syntax

We used searches of the internet, grey literature resources, and bibliographic databases to build out combinations of terms that would capture various aspects of each of these two broad types of humanitarian context. We did not assess the quality of the work that we identified, but rather looked only for potentially eligible search or indexing terms. We informally analysed terms used to describe settings in studies or editorials and we analysed the included studies in systematic reviews: the bibliographies of all were reviewed at title‐level, to look for new search terms.

This led to a draft structure of this cluster, which is illustrated in Figure 2, alongside a search narrative [8]. Here we list not only the detailed search syntax but also the resources/papers which informed and developed the search syntax.

### 2.3 Our final bibliographic search strategy

The final bibliographic search strategy is reported in Figure 3; this was our solution to the case. It is a refined version of approach 1b with the refinement coming in the work undertaken on the humanitarian cluster of terms. As Table 3 illustrates, whilst yield has grown, a higher number of marker papers were returned offering confidence and a lower Number Needed to Read (NNR) metric. The specific detail on the case, the search concepts related to humanitarian settings and food‐related outcomes, with supporting notes are reported in Figure 2.

Figure 3The final search strategy.

**Figure:**
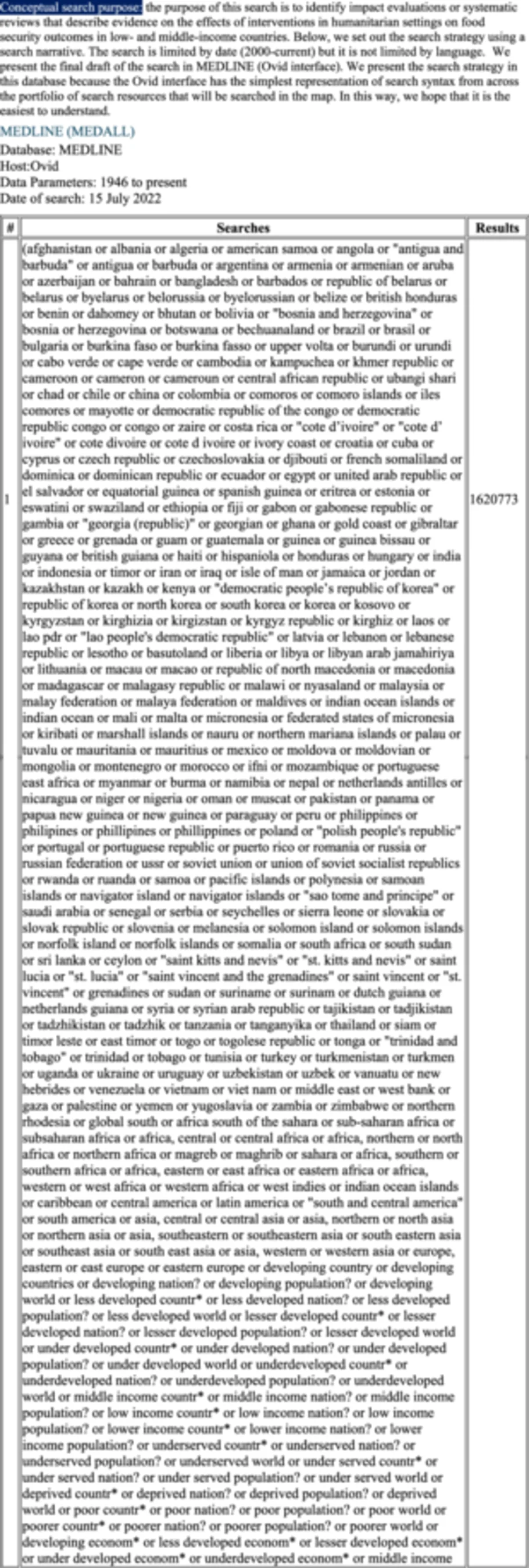

**Figure:**
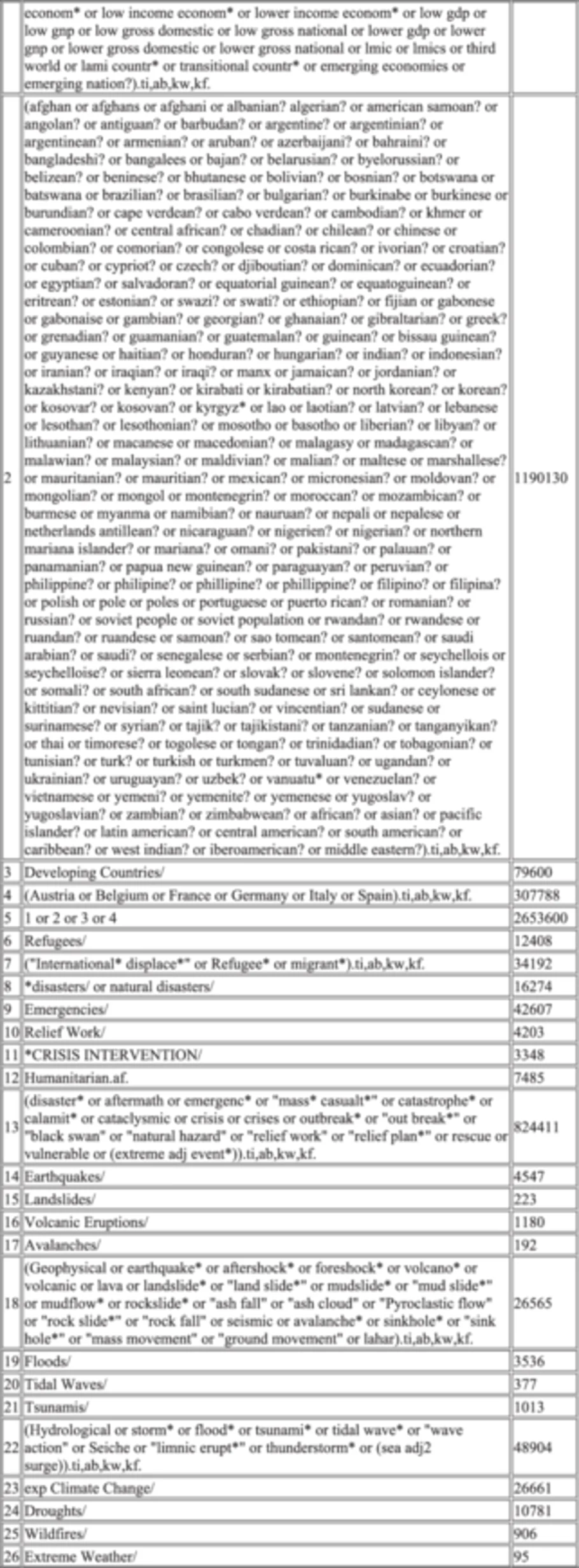

**Figure:**
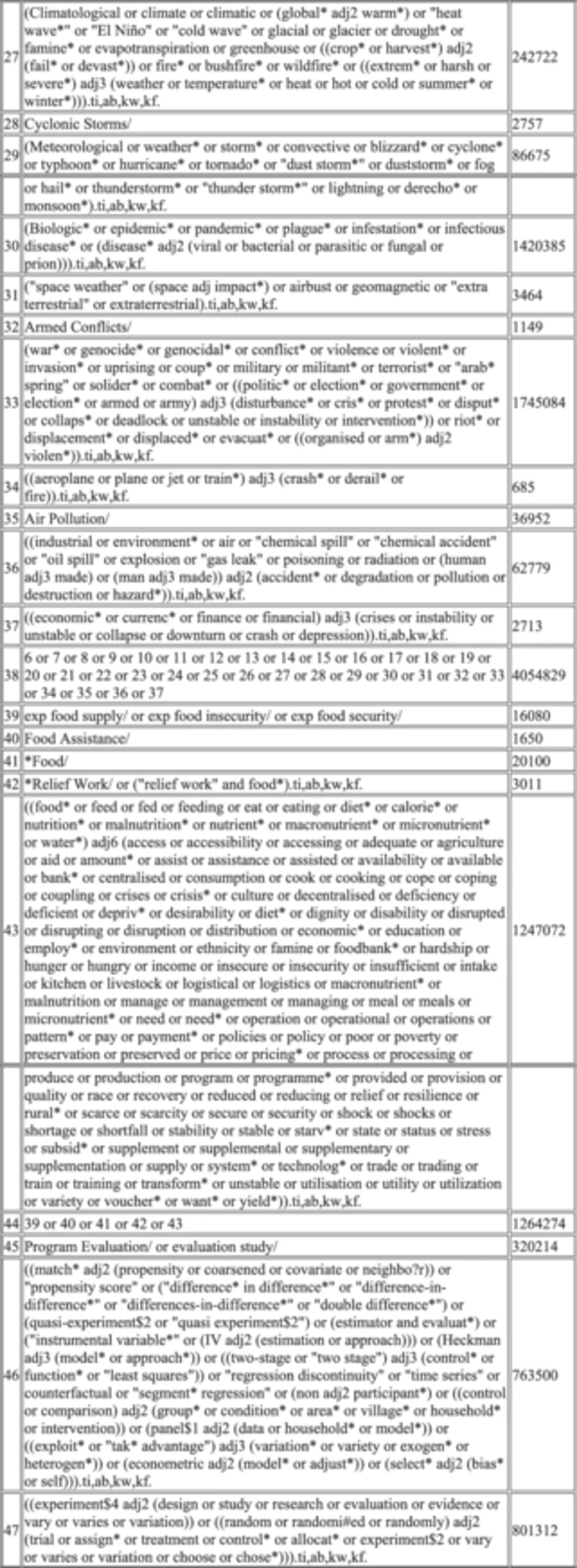

**Figure:**
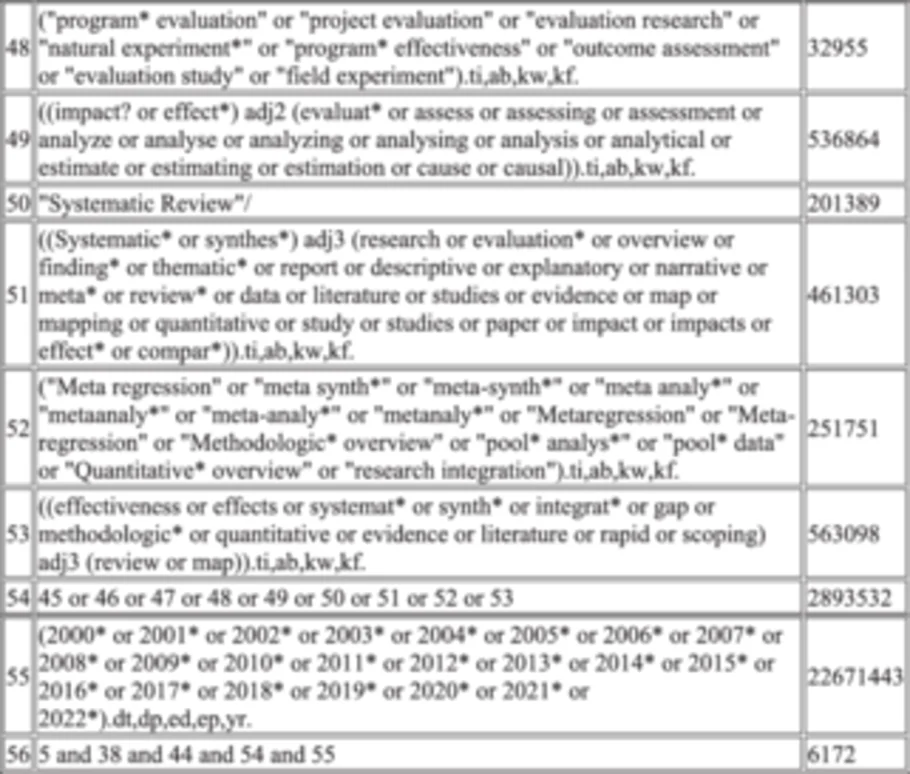

We anticipate that the search syntax in Figure 2 (taken from our MEDLINE search, Ovid interface) can be adopted for reviews which focus on humanitarian settings and translated to other databases as needed. We plan to report this section to the InterTASC Information Specialists' Sub‐Group Search Filter Resource [16]. Our hope is that by fully reporting the definitions and contexts used to develop our search concepts, we have been transparent for future users.

## 3 REFLECTIONS ON THE USE OF A CASE REPORT

Our intention in this case report has been to illustrate a challenge (the case) and to set out how we developed and then resolved the challenge (the resolution). We include detailed notes from our work and the final search strategy as a record of how a solution emerged from a multistep trial‐and‐error process. Below, we briefly elaborate the context of this report and the next steps for the idea.

*Is the idea for the use of case reports in evidence synthesis really novel?*

As we see it, these ‘case reports’ would differ from other forms of reporting evidence synthesis challenges. For example, a case report in evidence synthesis would be different to a tutorial, which aims to guide a researcher through a problem with an established procedure, and different to research in brief or a brief report, which describes or critiques a method of evidence synthesis or reports preliminary research findings [17, 18].

As we perceive it, and as we have set it out above, our idea for evidence synthesis case reports is for a simple and brief framing of a case and then a worked example of the solution, including the presentation of solutions and ideas which were explored but rejected. Our solution, in this instance, balanced sensitivity and feasibility given the context, timeline and resources we and the full EGM team were working with. Given that there is no one correct solution to this challenge, an idea we ultimately rejected could be an ideal solution for a similar case. The inclusion of rejected ideas in a case report provides educational value and potential ideas for further exploration. We believe this to be both novel and useful.

*How might these case reports be used?*

As our case illustrates, a considerable amount of unseen work goes into developing a search strategy [19, 20]. We envision that case reports like ours will offer a head‐start to future researchers working on similar problems. The work described in case reports might be adopted, adapted, or used to inform an entirely new search approach. Through publication of a case report, future researchers at least have the benefit of prior learning, even if they reject it going forward.

Whilst publication of a case report is likely to be reflective, after the synthesis has been completed and reported, methodological research offers many examples of a published exchange of letters between authors, which seek to critique and work together to further refine a problem (c.f. [21, 22, 23]). This might illustrate an opportunity for the community collaborate on a case, by transparent peer‐review and well intentioned critique. We would hope for this going forward as a general commentary on cases and reports.

The scalability of the singular report might represent a barrier to uptake. A report of a single example, even if clearly presented and supported by a detailed report, might still not convince all users. We acknowledge this potential limitation suggesting that, if adopted, case reports include a plan for dissemination (to ensure the case reaches its audience) and ownership by the original authors of the case's solution. In our instance, we propose to report our search to the ISSG search filters resource, so it is accessible, and we would be happy to hear from researchers to discuss our work (especially the feasibility of case reports generally and methodological problems specifically).

*The potential benefit of a separate publication and the expert voice*

Our proposal is that the case report as a form of report could be used for novel and unusual methodological problems of any type and in any discipline linked to evidence synthesis. These case reports might illustrate issues that are critical to the needs and practice of the methodologist, but are of little interest or felicity to the topic expert or decision‐maker. We therefore see these reports as separate publications, focused on a specific problem and readership, though they might—as here—speak across methodological disciplines.

Case reports, as separate from reports of a synthesis project's findings, allow for a detailed account of the case, which might not be possible to include with the main report within the word count of a journal article. Our sense is that detailing what did not work – or ideas tested but not pursued – is almost as important as setting out the resolution. However, the learning gained through exploring different approaches is rarely reflected in research reports that focus on the (ultimate) methods used and the results. If the aim of publication is to increase knowledge sharing and reduce research waste, then a transparent account of the workings to a solution may save time and reduce duplicated effort.

A further point in favour of case reports is that researchers who are processing a challenge, can relate their processes and conclusions in suitably technical language, since the case report is likely to be read by those with similar knowledge and interests. This will help reduce word count and the need for explanation of commonly agreed principles. In short, the case report can be kept short, as it might dispense preamble.

*Who would read these case reports?*

We see three potential audiences for this report specifically:

- **Individuals responsible for conducting the searches** (meaning information specialists/librarians/anyone charged with the identification of studies for reviews): this study reports specific detail on the development of a search strategy with granular detail on approaches tried and rejected and then on how we structured and developed the final search. Our work can be adopted or adapted for future searches. It can also be used for training purposes for newer searchers who have developed baseline search skills but lack experience with more complex searches and therefore wish to understand the thought process of an experienced searcher in solving this type of search challenge.
- **Researchers and people undertaking evidence synthesis**: the process of conceptualising and defining fairly abstract concepts (like “humanitarian context”) described in this report can help support future syntheses that attempt to encapsulate the state of scientific knowledge about similarly abstract concepts.
- **Decision makers**: review‐commissioning editors, funders, and people who work with evidence, might use this report to conceptualise the definitions and framing of humanitarian contexts, or to work within an aspect of this area (e.g., a a specific sub‐group).

Generally, though, we think case reports will usually speak most directly to a specific group of methodologists, depending on the case presented and the technicality of the resolution.

*But does the solution work?*

A question that naturally will arise is this: does the solution work? We would not consider it necessary for a case report to include an evaluation of effect or provide validation within the report. This, simply, because the case report is the report of a single case, and the case report itself really should only exist where the case is sufficiently novel to limit comparability, in its details, with other cases. This is not to say that the work here (or in future case reports) might not be subjected to evaluation or validation more generally in time.

We note, too, that whilst these ‘challenges’ may be unique, they do not often have one right solution: different practitioners could solve the problem in different ways. The design and testing of potential solutions itself represent a form of inquiry. Furthermore, there is a lack of established curricula and educational content for advanced skills training for some elements of evidence synthesis, including searching. The form of case report described above could therefore be used as an educational tool for improving the skills of students and new practitioners [24].

We mention this to be clear that the case report should be a simple and brief report of the case and the attempt to resolve the case. Researchers will have to take the solution at face value when determining if they will adopt, or how they will use, the resolution presented, remembering that the case was identified, and the solution developed, whilst the research was on‐going. This indicates, too, that case notes might also include reflections or notes containing insights gained with the benefit of hindsight.

## 4 CONCLUSIONS AND NEXT STEPS

We propose the adoption of case reports in evidence synthesis to detail complex problems that arise when undertaking complicated evidence synthesis, to report the solutions considered (including those not adopted), and to present the resolution ultimately adopted.

We illustrate an example case report for a complicated search strategy which sought to define then search for impact evaluations and systematic reviews focused on interventions in humanitarian contexts.

We welcome feedback on the idea for the use of case studies in evidence synthesis and any discussion on the structuring, dissemination, or use of these reports.

## AUTHOR CONTRIBUTIONS

**Chris Cooper**: Conceptualization, Data Curation, Investigation, Methodology, Project Administration, Writing – Original Draft Preparation, Writing – Review & Editing. **Zahra Premji**: Conceptualization, Data Curation, Investigation, Methodology, Project Administration, Writing – Original Draft Preparation, Writing – Review & Editing. **Cem Yavuz**: Conceptualization, Data Curation, Project Administration, Supervision, Writing – Review & Editing. **Mark Engelbert**: Conceptualization, Project Administration, Supervision, Writing – Review & Editing.

## CONFLICTS OF INTEREST

The authors declare no conflicts of interest.

## Supporting information

Supplementary information.

Supplementary information.

Supplementary information.

Supplementary information.

Supplementary information.

Supplementary information.

## Data Availability

All data relied upon is provided in the paper.
