# Supplementary material for: Should we adopt the case report format to report challenges in complicated evidence synthesis? A proposal and illustration of a case report of a complex search strategy for humanitarian interventions
Source: Cochrane Evid Synth Methods. 2025 Apr 13;3(3):e70021. doi: 10.1002/cesm.70021 (PMC12245084; doi:10.1002/cesm.70021)
Supplement: Supplementary file 3 — Supplementary information. [file CESM-3-e70021-s006.doc]

#3a humanitarian AND intervention AND food (broad)

| Database: Ovid MEDLINE(R) ALL <1946 to June 29, 2022>  Search Strategy: |  |
| --- | --- |
| 1 (afghanistan or albania or algeria or american samoa or angola or "antigua and barbuda" or antigua or barbuda or argentina or armenia or armenian or aruba or azerbaijan or bahrain or bangladesh or barbados or republic of belarus or belarus or byelarus or belorussia or byelorussian or belize or british honduras or benin or dahomey or bhutan or bolivia or "bosnia and herzegovina" or bosnia or herzegovina or botswana or bechuanaland or brazil or brasil or bulgaria or burkina faso or burkina fasso or upper volta or burundi or urundi or cabo verde or cape verde or cambodia or kampuchea or khmer republic or cameroon or cameron or cameroun or central african republic or ubangi shari or chad or chile or china or colombia or comoros or comoro islands or iles comores or mayotte or democratic republic of the congo or democratic republic congo or congo or zaire or costa rica or "cote d’ivoire" or "cote d’ ivoire" or cote divoire or cote d ivoire or ivory coast or croatia or cuba or cyprus or czech republic or czechoslovakia or djibouti or french somaliland or dominica or dominican republic or ecuador or egypt or united arab republic or el salvador or equatorial guinea or spanish guinea or eritrea or estonia or eswatini or swaziland or ethiopia or fiji or gabon or gabonese republic or gambia or "georgia (republic)" or georgian or ghana or gold coast or gibraltar or greece or grenada or guam or guatemala or guinea or guinea bissau or guyana or british guiana or haiti or hispaniola or honduras or hungary or india or indonesia or timor or iran or iraq or isle of man or jamaica or jordan or kazakhstan or kazakh or kenya or "democratic people’s republic of korea" or republic of korea or north korea or south korea or korea or kosovo or kyrgyzstan or kirghizia or kirgizstan or kyrgyz republic or kirghiz or laos or lao pdr or "lao people's democratic republic" or latvia or lebanon or lebanese republic or lesotho or basutoland or liberia or libya or libyan arab jamahiriya or lithuania or macau or macao or republic of north macedonia or macedonia or madagascar or malagasy republic or malawi or nyasaland or malaysia or malay federation or malaya federation or maldives or indian ocean islands or indian ocean or mali or malta or micronesia or federated states of micronesia or kiribati or marshall islands or nauru or northern mariana islands or palau or tuvalu or mauritania or mauritius or mexico or moldova or moldovian or mongolia or montenegro or morocco or ifni or mozambique or portuguese east africa or myanmar or burma or namibia or nepal or netherlands antilles or nicaragua or niger or nigeria or oman or muscat or pakistan or panama or papua new guinea or new guinea or paraguay or peru or philippines or philipines or phillipines or phillippines or poland or "polish people's republic" or portugal or portuguese republic or puerto rico or romania or russia or russian federation or ussr or soviet union or union of soviet socialist republics or rwanda or ruanda or samoa or pacific islands or polynesia or samoan islands or navigator island or navigator islands or "sao tome and principe" or saudi arabia or senegal or serbia or seychelles or sierra leone or slovakia or slovak republic or slovenia or melanesia or solomon island or solomon islands or norfolk island or norfolk islands or somalia or south africa or south sudan or sri lanka or ceylon or "saint kitts and nevis" or "st. kitts and nevis" or saint lucia or "st. lucia" or "saint vincent and the grenadines" or saint vincent or "st. vincent" or grenadines or sudan or suriname or surinam or dutch guiana or netherlands guiana or syria or syrian arab republic or tajikistan or tadjikistan or tadzhikistan or tadzhik or tanzania or tanganyika or thailand or siam or timor leste or east timor or togo or togolese republic or tonga or "trinidad and tobago" or trinidad or tobago or tunisia or turkey or turkmenistan or turkmen or uganda or ukraine or uruguay or uzbekistan or uzbek or vanuatu or new hebrides or venezuela or vietnam or viet nam or middle east or west bank or gaza or palestine or yemen or yugoslavia or zambia or zimbabwe or northern rhodesia or global south or africa south of the sahara or sub-saharan africa or subsaharan africa or africa, central or central africa or africa, northern or north africa or northern africa or magreb or maghrib or sahara or africa, southern or southern africa or africa, eastern or east africa or eastern africa or africa, western or west africa or western africa or west indies or indian ocean islands or caribbean or central america or latin america or "south and central america" or south america or asia, central or central asia or asia, northern or north asia or northern asia or asia, southeastern or southeastern asia or south eastern asia or southeast asia or south east asia or asia, western or western asia or europe, eastern or east europe or eastern europe or developing country or developing countries or developing nation? or developing population? or developing world or less developed countr* or less developed nation? or less developed population? or less developed world or lesser developed countr* or lesser developed nation? or lesser developed population? or lesser developed world or under developed countr* or under developed nation? or under developed population? or under developed world or underdeveloped countr* or underdeveloped nation? or underdeveloped population? or underdeveloped world or middle income countr* or middle income nation? or middle income population? or low income countr* or low income nation? or low income population? or lower income countr* or lower income nation? or lower income population? or underserved countr* or underserved nation? or underserved population? or underserved world or under served countr* or under served nation? or under served population? or under served world or deprived countr* or deprived nation? or deprived population? or deprived world or poor countr* or poor nation? or poor population? or poor world or poorer countr* or poorer nation? or poorer population? or poorer world or developing econom* or less developed econom* or lesser developed econom* or under developed econom* or underdeveloped econom* or middle income econom* or low income econom* or lower income econom* or low gdp or low gnp or low gross domestic or low gross national or lower gdp or lower gnp or lower gross domestic or lower gross national or lmic or lmics or third world or lami countr* or transitional countr* or emerging economies or emerging nation?).ti,ab,jn,kf. (1637941)  2 (afghan or afghans or afghani or albanian? algerian? or american samoan? or angolan? or antiguan? or barbudan? or argentine? or argentinian? or argentinean? or armenian? or aruban? or azerbaijani? or bahraini? or bangladeshi? or bangalees or bajan? or belarusian? or byelorussian? or belizean? or beninese? or bhutanese or bolivian? or bosnian? or botswana or batswana or brazilian? or brasilian? or bulgarian? or burkinabe or burkinese or burundian? or cape verdean? or cabo verdean? or cambodian? or khmer or cameroonian? or central african? or chadian? or chilean? or chinese or colombian? or comorian? or congolese or costa rican? or ivorian? or croatian? or cuban? or cypriot? or czech? or djiboutian? or dominican? or ecuadorian? or egyptian? or salvadoran? or equatorial guinean? or equatoguinean? or eritrean? or estonian? or swazi? or swati? or ethiopian? or fijian or gabonese or gabonaise or gambian? or georgian? or ghanaian? or gibraltarian? or greek? or grenadian? or guamanian? or guatemalan? or guinean? or bissau guinean? or guyanese or haitian? or honduran? or hungarian? or indian? or indonesian? or iranian? or iraqian? or iraqi? or manx or jamaican? or jordanian? or kazakhstani? or kenyan? or kirabati or kirabatian? or north korean? or korean? or kosovar? or kosovan? or kyrgyz* or lao or laotian? or latvian? or lebanese or lesothan? or lesothonian? or mosotho or basotho or liberian? or libyan? or lithuanian? or macanese or macedonian? or malagasy or madagascan? or malawian? or malaysian? or maldivian? or malian? or maltese or marshallese? or mauritanian? or mauritian? or mexican? or micronesian? or moldovan? or mongolian? or mongol or montenegrin? or moroccan? or mozambican? or burmese or myanma or namibian? or nauruan? or nepali or nepalese or netherlands antillean? or nicaraguan? or nigerien? or nigerian? or northern mariana islander? or mariana? or omani? or pakistani? or palauan? or panamanian? or papua new guinean? or paraguayan? or peruvian? or philippine? or philipine? or phillipine? or phillippine? or filipino? or filipina? or polish or pole or poles or portuguese or puerto rican? or romanian? or russian? or soviet people or soviet population or rwandan? or rwandese or ruandan? or ruandese or samoan? or sao tomean? or santomean? or saudi arabian? or saudi? or senegalese or serbian? or montenegrin? or seychellois or seychelloise? or sierra leonean? or slovak? or slovene? or solomon islander? or somali? or south african? or south sudanese or sri lankan? or ceylonese or kittitian? or nevisian? or saint lucian? or vincentian? or sudanese or surinamese? or syrian? or tajik? or tajikistani? or tanzanian? or tanganyikan? or thai or timorese? or togolese or tongan? or trinidadian? or tobagonian? or tunisian? or turk? or turkish or turkmen? or tuvaluan? or ugandan? or ukrainian? or uruguayan? or uzbek? or vanuatu* or venezuelan? or vietnamese or yemeni? or yemenite? or yemenese or yugoslav? or yugoslavian? or zambian? or zimbabwean? or african? or asian? or pacific islander? or latin american? or central american? or south american? or caribbean? or west indian? or iberoamerican? or middle eastern?).ti,ab,jn,kf. (1203490)  3 Refugees/ (12357)  4 (Refugee* or migrant*).ti,ab,kw,kf. (34767)  5 1 or 2 or 3 or 4 (2407985) | 3ie LMIC cluster (with additional terms for refugees per review inclusion criteria (for refugee camps) |
| 6 exp Disasters/ (95896)  7 Emergencies/ (42570)  8 exp Relief Work/ (5830)  9 CRISIS INTERVENTION/ (6054)  10 Humanitarian.af. (7696)  11 (disaster* or emergenc* or "mass* casualt*" or catastrophe* or calamit* or cataclysmic or crisis or crises or outbreak* or "out break*").ti,ab,kw,kf. (672562)  12 Earthquakes/ (4536)  13 Landslides/ (221)  14 Volcanic Eruptions/ (1173)  15 Avalanches/ (191)  16 (Geophysical or earthquake* or aftershock* or foreshock* or volcano* or volcanic or lava or landslide* or "land slide*" or mudslide* or mudflow* or avalanche* or sinkhole* or "sink hole*").ti,ab,kw,kf. (24648)  17 Floods/ (3523)  18 Tidal Waves/ (377)  19 Tsunamis/ (1008)  20 (Hydrological or strom* or flood* or tsunami* or tidal wave* or "limnic erupt*" or thunderstorm*).ti,ab,kw,kf. (168822)  21 exp Climate Change/ (26575)  22 Droughts/ (10752)  23 Wildfires/ (899)  24 Extreme Weather/ (94)  25 (Climatological or climate or climatic or (global* adj2 warm*) or "heat wave*" or drought* or famine* or fire* or bushfire* or wildfire* or ((extrem* or harsh or severe*) adj3 (weather or temperature* or heat or hot or cold or summer* or winter*))).ti,ab,kw,kf. (217039)  26 Cyclonic Storms/ (2744)  27 (Meteorological or weather* or storm* or blizzard* or cyclone* or typhoon* or hurricane* or tornado* or dust storm* or duststrom*).ti,ab,kw,kf. (67744)  28 (Biologic* or epidemic* or pandemic* or plague* or infestation* or infectious disease*).ti,ab,kw,kf. (1397227)  29 Armed Conflicts/ (1151)  30 (war* or genocide* or genocidal* or conflict* or violence or violent* or invasion* or uprising or coup* or military or militant* or terrorist* or "arab* spring" or ((politic* or election* or government* or election*) adj3 (disturbance* or cris* or protest* or disput* or collaps* deadlock)) or riot* or displacement* or displaced* or evacuat* or ((organised or arm*) adj2 violen*)).ti,ab,kw,kf. (1718801)  31 ((aeroplane or plane or jet or train*) adj3 (crash* or derail* or fire)).ti,ab,kw,kf. (689)  32 Air Pollution/ (36873)  33 ((industrial or environment* or air or (human adj3 made) or (man adj3 made)) adj2 (accident* or degradation or pollution or destruction or hazard*)).ti,ab,kw,kf. (58681)  34 ((economic* or currenc*) adj3 (crises or instability or unstable or collapse)).ti,ab,kw,kf. (1009)  35 6 or 7 or 8 or 9 or 10 or 11 or 12 or 13 or 14 or 15 or 16 or 17 or 18 or 19 or 20 or 21 or 22 or 23 or 24 or 25 or 26 or 27 or 28 or 29 or 30 or 31 or 32 or 33 or 34 (3957221) | Humanitarian (settings cluster) |
| 36 ((early or rapid*) adj4 (warning or warned or alert* or alarm* or action* or respons* or monitor or monitoring or model or models or forecast* or "fore cast*" or notification* or notify)).ti,ab,kw,kf. (109787)  37 (warning system or warning scoring).ti,ab,kw,kf. (2508)  38 exp *Economics/ (344304)  39 (finance or financial or microfinance or micro finance or economic* or cash* or bank* or borrowing or borrow or loan or loans or cost* or income* or capital or buy or buying or sell or selling or poverty or destitute or destitution or saving or savings or credit or insurance or insurable or insured or "risk shar*" or "risk transf*" or premium or overdraft or "over draft" or asset or assets or wage or income or salary or (social adj3 (support or safety))).ti,ab,kw,kf. (1447869)  40 (cash or cashless or voucher* or ration* or "in-kind" or transfer or gift* or cheque* or economy or "non-food" or stamp or stamps or barter or exchange or credit*).ti,ab,kw,kf. (1050688)  41 exp Agriculture/ (115578)  42 Livestock/ (4941)  43 (Agriculture or (Agri adj1 culture) or livestock or animal or animals or crop* or seeds or farm* or (food adj2 produc*) or technology or infrastructure or feed or grain or grazing).ti,ab,kw,kf. (2007154)  44 Nutrition Therapy/ or exp Malnutrition/ (134072)  45 (nutrient* or nutrition* or nutritious or diet* or food* or feeding or supplement* or fortif* or vitamin* or healthy).ti,ab,kw,kf. (2756551)  46 (market* adj3 (based or recover* or connect* or infrastructure or function*)).ti,ab,kf,kw. (3333)  47 *water/ (67093)  48 drinking water/ (10399)  49 exp Water Supply/ (34742)  50 *Sanitation/ (4960)  51 *Water Purification/ (28238)  52 (Water or aqua or hydration or sanitation* or rainwater*).ti,ab,kw,kf. (933876)  53 36 or 37 or 38 or 39 or 40 or 41 or 42 or 43 or 44 or 45 or 46 or 47 or 48 or 49 or 50 or 51 or 52 (7312984) | This is the ‘interventions’ cluster, based on the work in the protocol. This obvs very DRAFT. |
| 54 exp Food Security/ (431)  55 exp Food Supply/ (16013)  56 Food Assistance/ (1636)  57 *Food/ (20090)  58 (Food* or feed* or nutrition* or calorie*).ti,ab,kw,kf. (1254757)  59 54 or 55 or 56 or 57 or 58 (1261384) | Broad Food – broad because line 58 is not adjacent to terms for food security. |
| 60 Program Evaluation/ or evaluation study/ (320200)  61 ((match* adj2 (propensity or coarsened or covariate or neighbo?r)) or "propensity score" or ("difference* in difference*" or "difference-in-difference*" or "differences-in-difference*" or "double difference*") or (quasi-experiment$2 or "quasi experiment$2") or (estimator and evaluat*) or ("instrumental variable*" or (IV adj2 (estimation or approach))) or (Heckman adj3 (model* or approach*)) or ((two-stage or "two stage") adj3 (control* or function* or "least squares")) or "regression discontinuity" or "time series" or counterfactual or "segment* regression" or (non adj2 participant*) or ((control or comparison) adj2 (group* or condition* or area* or village* or household* or intervention)) or (panel$1 adj2 (data or household* or model*)) or ((exploit* or "tak* advantage") adj3 (variation* or variety or exogen* or heterogen*)) or (econometric adj2 (model* or adjust*)) or (select* adj2 (bias* or self))).ti,ab,kw,kf. (773816)  62 ((experiment$4 adj2 (design or study or research or evaluation or evidence or vary or varies or variation)) or ((random or randomi#ed or randomly) adj2 (trial or assign* or treatment or control* or allocat* or experiment$2 or vary or varies or variation or choose or chose*))).ti,ab,kw,kf. (811427)  63 ("program* evaluation" or "project evaluation" or "evaluation research" or "natural experiment*" or "program* effectiveness" or "outcome assessment" or "evaluation study" or "field experiment").ti,ab,kw,kf. (33594)  64 ((impact? or effect*) adj2 (evaluat* or assess or assessing or assessment or analyze or analyse or analyzing or analysing or analysis or analytical or estimate or estimating or estimation or cause or causal)).ti,ab,kw,kf. (544836)  65 "Systematic Review"/ (200284)  66 ((Systematic* or synthes*) adj3 (research or evaluation* or overview or finding* or thematic* or report or descriptive or explanatory or narrative or meta* or review* or data or literature or studies or evidence or map or mapping or quantitative or study or studies or paper or impact or impacts or effect* or compar*)).ti,ab,kw,kf. (472928)  67 ("Meta regression" or "meta synth*" or "meta-synth*" or "meta analy*" or "metaanaly*" or "meta-analy*" or "metanaly*" or "Metaregression" or "Meta-regression" or "Methodologic* overview" or "pool* analys*" or "pool* data" or "Quantitative* overview" or "research integration").ti,ab,kw,kf. (258081)  68 ((effectiveness or effects or systemat* or synth* or integrat* or gap or methodologic* or quantitative or evidence or literature or rapid or scoping) adj3 (review or map)).ti,ab,kw,kf. (576473)  69 60 or 61 or 62 or 63 or 64 or 65 or 66 or 67 or 68 (2932143) | 3ie search filters for IE and SR |
| 70 (2000* or 2001* or 2002* or 2003* or 2004* or 2005* or 2006* or 2007* or 2008* or 2009* or 2010* or 2011* or 2012* or 2013* or 2014* or 2015* or 2016* or 2017* or 2018* or 2019* or 2020* or 2021* or 2022*).dt,dp,ed,ep,yr. (22935560)  71 5 and 35 and 53 and 59 and 69 and 70 (4167) | Logic to combine the search  LINE 70  5 = LMIC cluster  35 = Humanitarian cluster  53 = Interventions cluster  59 = Broad food cluster  69 = 3ie IE/SR filters |

The two records below are blocked by the broad food cluster

1.

Delivering water, sanitation and hygiene interventions to women and children in conflict settings: a systematic review.

Als D, Meteke S, Stefopulos M, Gaffey MF, Kamali M, Munyuzangabo M, Shah S, Jain RP, Radhakrishnan A, Siddiqui FJ, Ataullahjan A, Bhutta ZA

BMJ Global Health. 5(Suppl 1), 2020 07.

[Journal Article. Research Support, Non-U.S. Gov't. Systematic Review]

UI: 32641288

BACKGROUND: Access to safe water and sanitation facilities and the adoption of effective hygiene practices are fundamental to reducing maternal and child morbidity and mortality globally. In armed conflict settings, inadequate water, sanitation and hygiene (WASH) infrastructure poses major health risks for women and children. This review aimed to synthesise the existing information on WASH interventions being delivered to women and children in conflict settings in low-income and middle-income countries (LMICs) and to identify the personnel, sites and platforms being used to deliver such interventions.

METHODS: We conducted a systematic search for publications indexed in four databases, and grey literature was searched through the websites of humanitarian agencies and organisations. Eligible publications reported WASH interventions delivered to conflict-affected women or children. We extracted and synthesised information on intervention delivery characteristics, as well as barriers and facilitators.

RESULTS: We identified 58 eligible publications reporting on the delivery of WASH interventions, mostly in Sub-Saharan Africa. Non-Governmental Organization (NGO)/United Nations (UN) agency staff were reported to be involved in delivering interventions in 62% of publications, with the most commonly reported delivery site being community spaces (50%). Only one publication reported quantitative data on intervention effectiveness among women or children.

DISCUSSION: This review revealed gaps in the current evidence on WASH intervention delivery in conflict settings. Little information is available on the delivery of water treatment or environmental hygiene interventions, or about the sites and personnel used to deliver WASH interventions. Limited quantitative data on WASH intervention coverage or effectiveness with respect to women or children are important gaps, as multiple factors can affect how WASH services are accessed differently by women and men, and the hygiene needs of adolescent girls and boys differ; these factors must be taken into account when delivering interventions in conflict settings.

PROSPERO REGISTRATION NUMBER: CRD42019125221.

Copyright © Author(s) (or their employer(s)) 2020. Re-use permitted under CC BY. Published by BMJ.

Version ID

1

Status

MEDLINE

Author NameID

Shah, Shailja; ORCID: <https://orcid.org/0000-0003-2329-5027> Jain, Reena P; ORCID: <https://orcid.org/0000-0001-7004-5093>

Authors Full Name

Als, Daina, Meteke, Sarah, Stefopulos, Marianne, Gaffey, Michelle F, Kamali, Mahdis, Munyuzangabo, Mariella, Shah, Shailja, Jain, Reena P, Radhakrishnan, Amruta, Siddiqui, Fahad J, Ataullahjan, Anushka, Bhutta, Zulfiqar A

Institution

Als, Daina. Centre for Global Child Health, Hospital for Sick Children, Toronto, Ontario, Canada. Meteke, Sarah. Centre for Global Child Health, Hospital for Sick Children, Toronto, Ontario, Canada.

Stefopulos, Marianne. Centre for Global Child Health, Hospital for Sick Children, Toronto, Ontario, Canada.

Gaffey, Michelle F. Centre for Global Child Health, Hospital for Sick Children, Toronto, Ontario, Canada.

Kamali, Mahdis. Centre for Global Child Health, Hospital for Sick Children, Toronto, Ontario, Canada.

Munyuzangabo, Mariella. Centre for Global Child Health, Hospital for Sick Children, Toronto, Ontario, Canada.

Shah, Shailja. Centre for Global Child Health, Hospital for Sick Children, Toronto, Ontario, Canada.

Jain, Reena P. Centre for Global Child Health, Hospital for Sick Children, Toronto, Ontario, Canada.

Radhakrishnan, Amruta. Centre for Global Child Health, Hospital for Sick Children, Toronto, Ontario, Canada.

Siddiqui, Fahad J. Centre for Global Child Health, Hospital for Sick Children, Toronto, Ontario, Canada.

Siddiqui, Fahad J. Health Services and Systems Research, Duke-NUS Graduate Medical School, Singapore.

Ataullahjan, Anushka. Centre for Global Child Health, Hospital for Sick Children, Toronto, Ontario, Canada.

Bhutta, Zulfiqar A. Centre for Global Child Health, Hospital for Sick Children, Toronto, Ontario, Canada zulfiqar.bhutta@sickkids.ca.

Bhutta, Zulfiqar A. Center of Excellence in Women and Child Health, Aga Khan University, Karachi, Pakistan.

Year of Publication

2020

2.

Does 'Manna from Heaven' help? The role of cash transfers in disaster recovery-lessons from Fiji after Tropical Cyclone Winston.

Ivaschenko O, Doyle J, Kim J, Sibley J, Majoka Z

Disasters. 44(3):455-476, 2020 Jul.

[Journal Article]

UI: 31583740

This paper contributes to the evidence on the effectiveness of shock-responsive social protection systems in helping affected households recover from the negative consequences of disasters. It evaluates the influence of the top-up cash transfers provided by the Government of Fiji to poor households in the wake of Tropical Cyclone Winston, which struck the Pacific Island country on 20 February 2016. The impact evaluation strategy incorporates a sharp regression discontinuity design to define treatment and control groups, based on the eligibility threshold of the poverty benefit scheme. The results indicate that treatment households-that is, those that received cash transfers-are significantly more likely to report quicker recovery from various shocks. Female-headed households are more likely to recover from the ramifications, whereas households with older heads are less likely to do so. The presence of a functioning market appears to be a major factor aiding the speed of recovery. Finally, the evidence points towards strong district effects on recovery.

Copyright © 2019 The Authors Disasters © 2019 Overseas Development Institute.

Version ID

1

Status

MEDLINE

Authors Full Name

Ivaschenko, Oleksiy, Doyle, Jesse, Kim, Jaekyun, Sibley, Jonathan, Majoka, Zaineb

Institution

Ivaschenko, Oleksiy. Senior Economist at the World Bank, United States. Doyle, Jesse. Economist at the World Bank, United States.

Kim, Jaekyun. Consultant at the World Bank, United States.

Sibley, Jonathan. Consultant at the World Bank, United States.

Majoka, Zaineb. Consultant at the World Bank, United States.

Year of Publication

2020

The five records below are blocked by the humanitarian cluster

3.

Food Security and Nutrition Outcomes of Farmer Field Schools in Eastern Democratic Republic of the Congo.

Doocy S, Cohen S, Emerson J, Menakuntuala J, Jenga Jamaa II Study Team, Santos Rocha J

Global Health Science & Practice. 5(4):630-643, 2017 12 28.

[Journal Article. Research Support, Non-U.S. Gov't. Research Support, U.S. Gov't, Non-P.H.S.]

UI: 29284698

BACKGROUND: Food and nutrition security in eastern Democratic Republic of the Congo are threatened by political instability and chronic poverty. The Jenga Jamaa II project, implemented between 2011 and 2016 in South Kivu Province, aimed to improve household food security and child nutritional status using various intervention strategies, including farmer field school (FFS) programs.

OBJECTIVE: To characterize the changes in agricultural production techniques, household food security, and child nutritional status associated with participation in FFS programs.

METHODS: We used a community-matched design to select FFS intervention and control households from 3 health zones in which the project was operating. Data on food security (Household Dietary Diversity Score [HDDS] and Household Food Insecurity Access Scale [HFIAS]) and child anthropometry were collected semiannually for 3.5 years in both groups. Additional data on agricultural practices were collected annually in the FFS group only. Focus groups with FFS staff and beneficiaries were conducted in the final project year. Statistical analyses included basic descriptive statistics such as paired t tests and analysis of covariance; regression models using a bootstrap were applied to generate P values and confidence intervals while accounting for differences between groups.

RESULTS: The study enrolled 388 FFS beneficiaries and their households in the intervention group and 324 non-FFS households in the control group. FFS participants reported increasing the number of different agricultural techniques they used by an average of 2.7 techniques over the project period, from 5.1 in 2013 to 7.9 in 2016 (P<.001). The mean HDDS and HFIAS improved more in the FFS group than in the control group (mean difference between intervention and control for HDDS was 0.9 points and for HFIAS was -4.6 points; P<.001). However, the prevalence of child stunting (60.2% intervention vs. 58.8% control) and underweight (22.3% intervention vs. 29.8% control) were similar in both groups at endline (P>.05).

CONCLUSION: Although FFS participants diversified their agricultural production strategies and experienced improvements in household food security, there was not a positive impact on child nutritional status. In this food-insecure context, improvements in agricultural production alone are unlikely to significantly change child nutritional status-a health outcome with a complex, multilevel causal chain.

Copyright © Doocy et al.

Version ID

1

Status

MEDLINE

Authors Full Name

Doocy, Shannon, Cohen, Sarah, Emerson, Jillian, Menakuntuala, Joseph, Jenga Jamaa II Study Team, Santos Rocha, Jozimo

Institution

Doocy, Shannon. Johns Hopkins Bloomberg School of Public Health, Baltimore, MD, USA. doocy1@jhu.edu. Cohen, Sarah. Johns Hopkins Bloomberg School of Public Health, Baltimore, MD, USA.

Emerson, Jillian. Johns Hopkins Bloomberg School of Public Health, Baltimore, MD, USA.

Menakuntuala, Joseph. Adventist Development and Relief Organization, Silver Spring, MD, USA.

Santos Rocha, Jozimo. Adventist Development and Relief Organization, Silver Spring, MD, USA.

Year of Publication

2017

4.

Planning an integrated agriculture and health program and designing its evaluation: Experience from Western Kenya.

Cole DC, Levin C, Loechl C, Thiele G, Grant F, Girard AW, Sindi K, Low J

Evaluation & Program Planning. 56:11-22, 2016 06.

[Journal Article. Research Support, Non-U.S. Gov't]

UI: 27003730

Multi-sectoral programs that involve stakeholders in agriculture, nutrition and health care are essential for responding to nutrition problems such as vitamin A deficiency among pregnant and lactating women and their infants in many poor areas of lower income countries. Yet planning such multi-sectoral programs and designing appropriate evaluations, to respond to different disciplinary cultures of evidence, remain a challenge. We describe the context, program development process, and evaluation design of the Mama SASHA project (Sweetpotato Action for Security and Health in Africa) which promoted production and consumption of a bio-fortified, orange-fleshed sweetpotato (OFSP). In planning the program we drew upon information from needs assessments, stakeholder consultations, and a first round of the implementation evaluation of a pilot project. The multi-disciplinary team worked with partner organizations to develop a program theory of change and an impact pathway which identified aspects of the program that would be monitored and established evaluation methods. Responding to the growing demand for greater rigour in impact evaluations, we carried out quasi-experimental allocation by health facility catchment area, repeat village surveys for assessment of change in intervention and control areas, and longitudinal tracking of individual mother-child pairs. Mid-course corrections in program implementation were informed by program monitoring, regular feedback from implementers and partners' meetings. To assess economic efficiency and provide evidence for scaling we collected data on resources used and project expenses. Managing the multi-sectoral program and the mixed methods evaluation involved bargaining and trade-offs that were deemed essential to respond to the array of stakeholders, program funders and disciplines involved.

Copyright © 2016 The Authors. Published by Elsevier Ltd.. All rights reserved.

Version ID

1

Status

MEDLINE

Authors Full Name

Cole, Donald C, Levin, Carol, Loechl, Cornelia, Thiele, Graham, Grant, Frederick, Girard, Aimee Webb, Sindi, Kirimi, Low, Jan

Institution

Cole, Donald C. Dalla Lana School of Public Health, University of Toronto, Toronto, Canada; International Potato Center (CIP), Peru. Electronic address: donald.cole@utoronto.ca. Levin, Carol. University of Washington, Seattle, USA.

Loechl, Cornelia. International Atomic Energy Agency, Vienna, Austria.

Thiele, Graham. CGIAR Research Program on Roots, Tubers and Bananas, Lima, Peru.

Grant, Frederick. CIP, Dar es Salaam, Tanzania.

Girard, Aimee Webb. Rollins School of Public Health, Emory University, GA, USA.

Sindi, Kirimi. CIP, Nairobi, Kenya.

Low, Jan. CIP, Nairobi, Kenya.

Year of Publication

2016

5.

School feeding, moving from practice to policy: reflections on building sustainable monitoring and evaluation systems.

Gelli A, Espejo F

Public Health Nutrition. 16(6):995-9, 2013 Jun.

[Journal Article]

UI: 22995677

OBJECTIVE: To provide an overview of the status of monitoring and evaluation (M&E) of school feeding across sub-Saharan Africa and to reflect on the experience on strengthening M&E systems to influence policy making in low-income countries.

DESIGN: Literature review on the M&E of school feeding programmes as well as data from World Food Programme surveys.

SETTING: Sub-Saharan Africa.

SUBJECTS: Countries implementing school feeding.

RESULTS: Only two randomized controlled impact evaluations have been implemented in sub-Saharan Africa. Where M&E data collection is underway, the focus is on process and service delivery and not on child outcomes. M&E systems generally operate under the Ministry of Education, with other Ministries represented within technical steering groups supporting implementation. There is no internationally accepted standardized framework for the M&E of school feeding. There have been examples where evidence of programme performance has influenced policy: considering the popularity of school feeding these cases though are anecdotal, highlighting the opportunity for systemic changes.

CONCLUSIONS: There is strong buy-in on school feeding from governments in sub-Saharan Africa. In response to this demand, development partners have been harmonizing their support to strengthen national programmes, with a focus on M&E. However, policy processes are complex and can be influenced by a number of factors. A comprehensive but simple approach is needed where the first step is to ensure a valid mandate to intervene, legitimizing the interaction with key stakeholders, involving them in the problem definition and problem solving. This process has been facilitated through the provision of technical assistance and exposure to successful experiences through South-South cooperation and knowledge exchange.

Version ID

1

Status

MEDLINE

Authors Full Name

Gelli, Aulo, Espejo, Francisco

Institution

Gelli, Aulo. The Partnership for Child Development, Department of Infectious Disease Epidemiology, Imperial College London W2 1PG, UK. aulo.gelli09@imperial.ac.uk

Year of Publication

2013

6.

An evaluation of an operations research project to reduce childhood stunting in a food-insecure area in Ethiopia.

Fenn B, Bulti AT, Nduna T, Duffield A, Watson F

Public Health Nutrition. 15(9):1746-54, 2012 Sep.

[Evaluation Study. Journal Article. Research Support, Non-U.S. Gov't]

UI: 22717058

OBJECTIVE: To determine which interventions can reduce linear growth retardation (stunting) in children aged 6-36 months over a 5-year period in a food-insecure population in Ethiopia.

DESIGN: We used data collected through an operations research project run by Save the Children UK: the Child Caring Practices (CCP) project. Eleven neighbouring villages were purposefully selected to receive one of four interventions: (i) health; (iii) nutrition education; (iii) water, sanitation and hygiene (WASH); or (iv) integrated comprising all interventions. A comparison group of three villages did not receive any interventions. Cross-sectional surveys were conducted at baseline (2004) and for impact evaluation (2009) using the same quantitative and qualitative tools. The primary outcome was stunted growth in children aged 6-36 months measured as height (or length)-for-age Z-scores (mean and prevalence). Secondary outcomes were knowledge of health seeking, infant and young child feeding and preventive practices.

SETTING: Amhara, Ethiopia.

SUBJECTS: Children aged 6-36 months.

RESULTS: The WASH intervention group was the only group to show a significant increase in mean height-for-age Z-score (+0.33, P = 0.02), with a 12.1 % decrease in the prevalence of stunting, compared with the baseline group. This group also showed significant improvements in mothers' knowledge of causes of diarrhoea and hygiene practices. The other intervention groups saw non-significant impacts for childhood stunting but improvements in knowledge relating to specific intervention education messages given.

CONCLUSIONS: The study suggests that an improvement in hygiene practices had a significant impact on stunting levels. However, there may be alternative explanations for this and further evidence is required.

Version ID

1

Status

MEDLINE

Authors Full Name

Fenn, Bridget, Bulti, Assaye T, Nduna, Themba, Duffield, Arabella, Watson, Fiona

Institution

Fenn, Bridget. Le Rocher, 61210 La Foret Auvray, France. fennysnake@gmail.com

Year of Publication

2012

7.

Impact of a cash-for-work programme on food consumption and nutrition among women and children facing food insecurity in rural Bangladesh.

Mascie-Taylor CG, Marks MK, Goto R, Islam R

Bulletin of the World Health Organization. 88(11):854-60, 2010 Nov 01.

[Journal Article. Research Support, Non-U.S. Gov't]

UI: 21076567

OBJECTIVE: To determine whether a cash-for-work programme during the annual food insecurity period in Bangladesh improved nutritional status in poor rural women and children.

METHODS: The panel study involved a random sample of 895 households from over 50,000 enrolled in a cash-for-work programme between September and December 2007 and 921 similar control households. The height, weight and mid-upper arm circumference of one woman and child aged less than 5 years from each household were measured at baseline and at the end of the study (mean time: 10 weeks). Women reported 7-day household food expenditure and consumption on both occasions. Changes in parameters were compared between the two groups.

FINDINGS: At baseline, no significant difference existed between the groups. By the study end, the difference in mean mid-upper arm circumference between women in the intervention and control groups had widened by 2.29 mm and the difference in mean weight, by 0.88 kg. Among children, the difference in means between the two groups had also widened in favour of the intervention group for: height (0.08 cm; P<0.05), weight (0.22 kg; P<0.001), mid-upper arm circumference (1.41 mm; P<0.001) and z-scores for height-for-age (0.02; P<0.001), weight-for-age (0.17; P<0.001), weight-for-height (0.23; P<0.001) and mid-upper arm circumference (0.12; P<0.001). Intervention households spent more on food and consumed more protein-rich food at the end of the study.

CONCLUSION: The cash-for-work programme led to greater household food expenditure and consumption and women's and children's nutritional status improved.

Version ID

1

Status

MEDLINE

Authors Full Name

Mascie-Taylor, C G N, Marks, M K, Goto, R, Islam, R

Institution

Mascie-Taylor, C G N. Department of Biological Anthropology, University of Cambridge, Pembroke Street, Cambridge, CB2 3RA, England. nmt1@cam.ac.uk

Year of Publication

2010
