# Supplementary material for: Should we adopt the case report format to report challenges in complicated evidence synthesis? A proposal and illustration of a case report of a complex search strategy for humanitarian interventions
Source: Cochrane Evid Synth Methods. 2025 Apr 13;3(3):e70021. doi: 10.1002/cesm.70021 (PMC12245084; doi:10.1002/cesm.70021)
Supplement: Supplementary file 4 — Supplementary information. [file CESM-3-e70021-s001.doc]

#3b humanitarian OR intervention AND food (focused)

| Database: Ovid MEDLINE(R) ALL <1946 to June 29, 2022>  Search Strategy: |  |
| --- | --- |
| 1 (afghanistan or albania or algeria or american samoa or angola or "antigua and barbuda" or antigua or barbuda or argentina or armenia or armenian or aruba or azerbaijan or bahrain or bangladesh or barbados or republic of belarus or belarus or byelarus or belorussia or byelorussian or belize or british honduras or benin or dahomey or bhutan or bolivia or "bosnia and herzegovina" or bosnia or herzegovina or botswana or bechuanaland or brazil or brasil or bulgaria or burkina faso or burkina fasso or upper volta or burundi or urundi or cabo verde or cape verde or cambodia or kampuchea or khmer republic or cameroon or cameron or cameroun or central african republic or ubangi shari or chad or chile or china or colombia or comoros or comoro islands or iles comores or mayotte or democratic republic of the congo or democratic republic congo or congo or zaire or costa rica or "cote d’ivoire" or "cote d’ ivoire" or cote divoire or cote d ivoire or ivory coast or croatia or cuba or cyprus or czech republic or czechoslovakia or djibouti or french somaliland or dominica or dominican republic or ecuador or egypt or united arab republic or el salvador or equatorial guinea or spanish guinea or eritrea or estonia or eswatini or swaziland or ethiopia or fiji or gabon or gabonese republic or gambia or "georgia (republic)" or georgian or ghana or gold coast or gibraltar or greece or grenada or guam or guatemala or guinea or guinea bissau or guyana or british guiana or haiti or hispaniola or honduras or hungary or india or indonesia or timor or iran or iraq or isle of man or jamaica or jordan or kazakhstan or kazakh or kenya or "democratic people’s republic of korea" or republic of korea or north korea or south korea or korea or kosovo or kyrgyzstan or kirghizia or kirgizstan or kyrgyz republic or kirghiz or laos or lao pdr or "lao people's democratic republic" or latvia or lebanon or lebanese republic or lesotho or basutoland or liberia or libya or libyan arab jamahiriya or lithuania or macau or macao or republic of north macedonia or macedonia or madagascar or malagasy republic or malawi or nyasaland or malaysia or malay federation or malaya federation or maldives or indian ocean islands or indian ocean or mali or malta or micronesia or federated states of micronesia or kiribati or marshall islands or nauru or northern mariana islands or palau or tuvalu or mauritania or mauritius or mexico or moldova or moldovian or mongolia or montenegro or morocco or ifni or mozambique or portuguese east africa or myanmar or burma or namibia or nepal or netherlands antilles or nicaragua or niger or nigeria or oman or muscat or pakistan or panama or papua new guinea or new guinea or paraguay or peru or philippines or philipines or phillipines or phillippines or poland or "polish people's republic" or portugal or portuguese republic or puerto rico or romania or russia or russian federation or ussr or soviet union or union of soviet socialist republics or rwanda or ruanda or samoa or pacific islands or polynesia or samoan islands or navigator island or navigator islands or "sao tome and principe" or saudi arabia or senegal or serbia or seychelles or sierra leone or slovakia or slovak republic or slovenia or melanesia or solomon island or solomon islands or norfolk island or norfolk islands or somalia or south africa or south sudan or sri lanka or ceylon or "saint kitts and nevis" or "st. kitts and nevis" or saint lucia or "st. lucia" or "saint vincent and the grenadines" or saint vincent or "st. vincent" or grenadines or sudan or suriname or surinam or dutch guiana or netherlands guiana or syria or syrian arab republic or tajikistan or tadjikistan or tadzhikistan or tadzhik or tanzania or tanganyika or thailand or siam or timor leste or east timor or togo or togolese republic or tonga or "trinidad and tobago" or trinidad or tobago or tunisia or turkey or turkmenistan or turkmen or uganda or ukraine or uruguay or uzbekistan or uzbek or vanuatu or new hebrides or venezuela or vietnam or viet nam or middle east or west bank or gaza or palestine or yemen or yugoslavia or zambia or zimbabwe or northern rhodesia or global south or africa south of the sahara or sub-saharan africa or subsaharan africa or africa, central or central africa or africa, northern or north africa or northern africa or magreb or maghrib or sahara or africa, southern or southern africa or africa, eastern or east africa or eastern africa or africa, western or west africa or western africa or west indies or indian ocean islands or caribbean or central america or latin america or "south and central america" or south america or asia, central or central asia or asia, northern or north asia or northern asia or asia, southeastern or southeastern asia or south eastern asia or southeast asia or south east asia or asia, western or western asia or europe, eastern or east europe or eastern europe or developing country or developing countries or developing nation? or developing population? or developing world or less developed countr* or less developed nation? or less developed population? or less developed world or lesser developed countr* or lesser developed nation? or lesser developed population? or lesser developed world or under developed countr* or under developed nation? or under developed population? or under developed world or underdeveloped countr* or underdeveloped nation? or underdeveloped population? or underdeveloped world or middle income countr* or middle income nation? or middle income population? or low income countr* or low income nation? or low income population? or lower income countr* or lower income nation? or lower income population? or underserved countr* or underserved nation? or underserved population? or underserved world or under served countr* or under served nation? or under served population? or under served world or deprived countr* or deprived nation? or deprived population? or deprived world or poor countr* or poor nation? or poor population? or poor world or poorer countr* or poorer nation? or poorer population? or poorer world or developing econom* or less developed econom* or lesser developed econom* or under developed econom* or underdeveloped econom* or middle income econom* or low income econom* or lower income econom* or low gdp or low gnp or low gross domestic or low gross national or lower gdp or lower gnp or lower gross domestic or lower gross national or lmic or lmics or third world or lami countr* or transitional countr* or emerging economies or emerging nation?).ti,ab,jn,kf. (1637941)  2 (afghan or afghans or afghani or albanian? algerian? or american samoan? or angolan? or antiguan? or barbudan? or argentine? or argentinian? or argentinean? or armenian? or aruban? or azerbaijani? or bahraini? or bangladeshi? or bangalees or bajan? or belarusian? or byelorussian? or belizean? or beninese? or bhutanese or bolivian? or bosnian? or botswana or batswana or brazilian? or brasilian? or bulgarian? or burkinabe or burkinese or burundian? or cape verdean? or cabo verdean? or cambodian? or khmer or cameroonian? or central african? or chadian? or chilean? or chinese or colombian? or comorian? or congolese or costa rican? or ivorian? or croatian? or cuban? or cypriot? or czech? or djiboutian? or dominican? or ecuadorian? or egyptian? or salvadoran? or equatorial guinean? or equatoguinean? or eritrean? or estonian? or swazi? or swati? or ethiopian? or fijian or gabonese or gabonaise or gambian? or georgian? or ghanaian? or gibraltarian? or greek? or grenadian? or guamanian? or guatemalan? or guinean? or bissau guinean? or guyanese or haitian? or honduran? or hungarian? or indian? or indonesian? or iranian? or iraqian? or iraqi? or manx or jamaican? or jordanian? or kazakhstani? or kenyan? or kirabati or kirabatian? or north korean? or korean? or kosovar? or kosovan? or kyrgyz* or lao or laotian? or latvian? or lebanese or lesothan? or lesothonian? or mosotho or basotho or liberian? or libyan? or lithuanian? or macanese or macedonian? or malagasy or madagascan? or malawian? or malaysian? or maldivian? or malian? or maltese or marshallese? or mauritanian? or mauritian? or mexican? or micronesian? or moldovan? or mongolian? or mongol or montenegrin? or moroccan? or mozambican? or burmese or myanma or namibian? or nauruan? or nepali or nepalese or netherlands antillean? or nicaraguan? or nigerien? or nigerian? or northern mariana islander? or mariana? or omani? or pakistani? or palauan? or panamanian? or papua new guinean? or paraguayan? or peruvian? or philippine? or philipine? or phillipine? or phillippine? or filipino? or filipina? or polish or pole or poles or portuguese or puerto rican? or romanian? or russian? or soviet people or soviet population or rwandan? or rwandese or ruandan? or ruandese or samoan? or sao tomean? or santomean? or saudi arabian? or saudi? or senegalese or serbian? or montenegrin? or seychellois or seychelloise? or sierra leonean? or slovak? or slovene? or solomon islander? or somali? or south african? or south sudanese or sri lankan? or ceylonese or kittitian? or nevisian? or saint lucian? or vincentian? or sudanese or surinamese? or syrian? or tajik? or tajikistani? or tanzanian? or tanganyikan? or thai or timorese? or togolese or tongan? or trinidadian? or tobagonian? or tunisian? or turk? or turkish or turkmen? or tuvaluan? or ugandan? or ukrainian? or uruguayan? or uzbek? or vanuatu* or venezuelan? or vietnamese or yemeni? or yemenite? or yemenese or yugoslav? or yugoslavian? or zambian? or zimbabwean? or african? or asian? or pacific islander? or latin american? or central american? or south american? or caribbean? or west indian? or iberoamerican? or middle eastern?).ti,ab,jn,kf. (1203490)  3 Refugees/ (12357)  4 (Refugee* or migrant*).ti,ab,kw,kf. (34767)  5 1 or 2 or 3 or 4 (2407985) | 3ie LMIC cluster (with additional terms for refugees per review inclusion criteria (for refugee camps) |
| 6 exp Disasters/ (95896)  7 Emergencies/ (42570)  8 exp Relief Work/ (5830)  9 CRISIS INTERVENTION/ (6054)  10 Humanitarian.af. (7696)  11 (disaster* or emergenc* or "mass* casualt*" or catastrophe* or calamit* or cataclysmic or crisis or crises or outbreak* or "out break*").ti,ab,kw,kf. (672562)  12 Earthquakes/ (4536)  13 Landslides/ (221)  14 Volcanic Eruptions/ (1173)  15 Avalanches/ (191)  16 (Geophysical or earthquake* or aftershock* or foreshock* or volcano* or volcanic or lava or landslide* or "land slide*" or mudslide* or mudflow* or avalanche* or sinkhole* or "sink hole*").ti,ab,kw,kf. (24648)  17 Floods/ (3523)  18 Tidal Waves/ (377)  19 Tsunamis/ (1008)  20 (Hydrological or strom* or flood* or tsunami* or tidal wave* or "limnic erupt*" or thunderstorm*).ti,ab,kw,kf. (168822)  21 exp Climate Change/ (26575)  22 Droughts/ (10752)  23 Wildfires/ (899)  24 Extreme Weather/ (94)  25 (Climatological or climate or climatic or (global* adj2 warm*) or "heat wave*" or drought* or famine* or fire* or bushfire* or wildfire* or ((extrem* or harsh or severe*) adj3 (weather or temperature* or heat or hot or cold or summer* or winter*))).ti,ab,kw,kf. (217039)  26 Cyclonic Storms/ (2744)  27 (Meteorological or weather* or storm* or blizzard* or cyclone* or typhoon* or hurricane* or tornado* or dust storm* or duststrom*).ti,ab,kw,kf. (67744)  28 (Biologic* or epidemic* or pandemic* or plague* or infestation* or infectious disease*).ti,ab,kw,kf. (1397227)  29 Armed Conflicts/ (1151)  30 (war* or genocide* or genocidal* or conflict* or violence or violent* or invasion* or uprising or coup* or military or militant* or terrorist* or "arab* spring" or ((politic* or election* or government* or election*) adj3 (disturbance* or cris* or protest* or disput* or collaps* deadlock)) or riot* or displacement* or displaced* or evacuat* or ((organised or arm*) adj2 violen*)).ti,ab,kw,kf. (1718801)  31 ((aeroplane or plane or jet or train*) adj3 (crash* or derail* or fire)).ti,ab,kw,kf. (689)  32 Air Pollution/ (36873)  33 ((industrial or environment* or air or (human adj3 made) or (man adj3 made)) adj2 (accident* or degradation or pollution or destruction or hazard*)).ti,ab,kw,kf. (58681)  34 ((economic* or currenc*) adj3 (crises or instability or unstable or collapse)).ti,ab,kw,kf. (1009)  35 6 or 7 or 8 or 9 or 10 or 11 or 12 or 13 or 14 or 15 or 16 or 17 or 18 or 19 or 20 or 21 or 22 or 23 or 24 or 25 or 26 or 27 or 28 or 29 or 30 or 31 or 32 or 33 or 34 (3957221) | Humanitarian (settings cluster) |
| 36 ((early or rapid*) adj4 (warning or warned or alert* or alarm* or action* or respons* or monitor or monitoring or model or models or forecast* or "fore cast*" or notification* or notify)).ti,ab,kw,kf. (109787)  37 (warning system or warning scoring).ti,ab,kw,kf. (2508)  38 exp *Economics/ (344304)  39 (finance or financial or microfinance or micro finance or economic* or cash* or bank* or borrowing or borrow or loan or loans or cost* or income* or capital or buy or buying or sell or selling or poverty or destitute or destitution or saving or savings or credit or insurance or insurable or insured or "risk shar*" or "risk transf*" or premium or overdraft or "over draft" or asset or assets or wage or income or salary or (social adj3 (support or safety))).ti,ab,kw,kf. (1447869)  40 (cash or cashless or voucher* or ration* or "in-kind" or transfer or gift* or cheque* or economy or "non-food" or stamp or stamps or barter or exchange or credit*).ti,ab,kw,kf. (1050688)  41 exp Agriculture/ (115578)  42 Livestock/ (4941)  43 (Agriculture or (Agri adj1 culture) or livestock or animal or animals or crop* or seeds or farm* or (food adj2 produc*) or technology or infrastructure or feed or grain or grazing).ti,ab,kw,kf. (2007154)  44 Nutrition Therapy/ or exp Malnutrition/ (134072)  45 (nutrient* or nutrition* or nutritious or diet* or food* or feeding or supplement* or fortif* or vitamin* or healthy).ti,ab,kw,kf. (2756551)  46 (market* adj3 (based or recover* or connect* or infrastructure or function*)).ti,ab,kf,kw. (3333)  47 *water/ (67093)  48 drinking water/ (10399)  49 exp Water Supply/ (34742)  50 *Sanitation/ (4960)  51 *Water Purification/ (28238)  52 (Water or aqua or hydration or sanitation* or rainwater*).ti,ab,kw,kf. (933876)  53 36 or 37 or 38 or 39 or 40 or 41 or 42 or 43 or 44 or 45 or 46 or 47 or 48 or 49 or 50 or 51 or 52 (7312984) | This is the ‘interventions’ cluster, based on the work in the protocol. |
| 54 exp Food Security/ (431)  55 exp Food Supply/ (16013)  56 Food Assistance/ (1636)  57 *Food/ (20090)  58 *Relief Work/ (3004)  59 ((food* or feed or fed or feeding or eat or eating or diet* or calorie* or nutrition* or malnutrition* or water*) adj6 (access or accessibility or accessing or adequate or assist or assistance or assisted or availability or available or bank* or consumption or cope or coping or crisis* or depriv* or desirability or diet* or disability disrupted or disrupting or disruption or economic* or employ* or ethnicity or foodbank* or hardship or hunger or hungry or income or insecure or insecurity or insufficient or intake or kitchen or macronutrient* or malnutrition or micronutrient* or need or need* or nutrition or pattern* or policies or policy or poor or poverty or poverty or produce or production or program or programme or provided or provision or quality or race or reduced or reducing or scarce or scarcity or secure or security or shortage or shortfall or stability or stable or starv* or state or status or supply or trade or trading or train or training unstable or utilisation or utility or utilization or variety or want*)).ti,ab,kf,kw. (1147641)  60 54 or 55 or 56 or 57 or 58 or 59 (1166003) | Focused food – this combines terms for food with terms for security within 5 spaces.  THIS IS UNDER DEVELOPMENT NOW. So DRAFT. |
| 61 Program Evaluation/ or evaluation study/ (320200)  62 ((match* adj2 (propensity or coarsened or covariate or neighbo?r)) or "propensity score" or ("difference* in difference*" or "difference-in-difference*" or "differences-in-difference*" or "double difference*") or (quasi-experiment$2 or "quasi experiment$2") or (estimator and evaluat*) or ("instrumental variable*" or (IV adj2 (estimation or approach))) or (Heckman adj3 (model* or approach*)) or ((two-stage or "two stage") adj3 (control* or function* or "least squares")) or "regression discontinuity" or "time series" or counterfactual or "segment* regression" or (non adj2 participant*) or ((control or comparison) adj2 (group* or condition* or area* or village* or household* or intervention)) or (panel$1 adj2 (data or household* or model*)) or ((exploit* or "tak* advantage") adj3 (variation* or variety or exogen* or heterogen*)) or (econometric adj2 (model* or adjust*)) or (select* adj2 (bias* or self))).ti,ab,kw,kf. (773816)  63 ((experiment$4 adj2 (design or study or research or evaluation or evidence or vary or varies or variation)) or ((random or randomi#ed or randomly) adj2 (trial or assign* or treatment or control* or allocat* or experiment$2 or vary or varies or variation or choose or chose*))).ti,ab,kw,kf. (811427)  64 ("program* evaluation" or "project evaluation" or "evaluation research" or "natural experiment*" or "program* effectiveness" or "outcome assessment" or "evaluation study" or "field experiment").ti,ab,kw,kf. (33594)  65 ((impact? or effect*) adj2 (evaluat* or assess or assessing or assessment or analyze or analyse or analyzing or analysing or analysis or analytical or estimate or estimating or estimation or cause or causal)).ti,ab,kw,kf. (544836)  66 "Systematic Review"/ (200284)  67 ((Systematic* or synthes*) adj3 (research or evaluation* or overview or finding* or thematic* or report or descriptive or explanatory or narrative or meta* or review* or data or literature or studies or evidence or map or mapping or quantitative or study or studies or paper or impact or impacts or effect* or compar*)).ti,ab,kw,kf. (472928)  68 ("Meta regression" or "meta synth*" or "meta-synth*" or "meta analy*" or "metaanaly*" or "meta-analy*" or "metanaly*" or "Metaregression" or "Meta-regression" or "Methodologic* overview" or "pool* analys*" or "pool* data" or "Quantitative* overview" or "research integration").ti,ab,kw,kf. (258081)  69 ((effectiveness or effects or systemat* or synth* or integrat* or gap or methodologic* or quantitative or evidence or literature or rapid or scoping) adj3 (review or map)).ti,ab,kw,kf. (576473)  70 61 or 62 or 63 or 64 or 65 or 66 or 67 or 68 or 69 (2932143) | 3ie search filters for IE and SR |
| 71 35 or 53 (10102020)  72 5 and 60 and 70 and 71 (24600)  73 (2000* or 2001* or 2002* or 2003* or 2004* or 2005* or 2006* or 2007* or 2008* or 2009* or 2010* or 2011* or 2012* or 2013* or 2014* or 2015* or 2016* or 2017* or 2018* or 2019* or 2020* or 2021* or 2022*).dt,dp,ed,ep,yr. (22935560)  74 72 and 73 (22481) | Search logic to complete the search  71 = humanitarian terms OR intervention terms  72 =  * 5 = LMIC  * 60 = focused food  * 70 = 3ie methods  * 71 = humanitarian terms OR intervention terms |

Studies were only blocked by the LMIC cluster, so not listed here.
