# Supplementary material for: Should we adopt the case report format to report challenges in complicated evidence synthesis? A proposal and illustration of a case report of a complex search strategy for humanitarian interventions
Source: Cochrane Evid Synth Methods. 2025 Apr 13;3(3):e70021. doi: 10.1002/cesm.70021 (PMC12245084; doi:10.1002/cesm.70021)
Supplement: Supplementary file 5 — Supplementary information. [file CESM-3-e70021-s003.doc]

#3c humanitarian OR intervention AND food (broad)

| Database: Ovid MEDLINE(R) ALL <1946 to June 29, 2022> |  |
| --- | --- |
| 1 (afghanistan or albania or algeria or american samoa or angola or "antigua and barbuda" or antigua or barbuda or argentina or armenia or armenian or aruba or azerbaijan or bahrain or bangladesh or barbados or republic of belarus or belarus or byelarus or belorussia or byelorussian or belize or british honduras or benin or dahomey or bhutan or bolivia or "bosnia and herzegovina" or bosnia or herzegovina or botswana or bechuanaland or brazil or brasil or bulgaria or burkina faso or burkina fasso or upper volta or burundi or urundi or cabo verde or cape verde or cambodia or kampuchea or khmer republic or cameroon or cameron or cameroun or central african republic or ubangi shari or chad or chile or china or colombia or comoros or comoro islands or iles comores or mayotte or democratic republic of the congo or democratic republic congo or congo or zaire or costa rica or "cote d’ivoire" or "cote d’ ivoire" or cote divoire or cote d ivoire or ivory coast or croatia or cuba or cyprus or czech republic or czechoslovakia or djibouti or french somaliland or dominica or dominican republic or ecuador or egypt or united arab republic or el salvador or equatorial guinea or spanish guinea or eritrea or estonia or eswatini or swaziland or ethiopia or fiji or gabon or gabonese republic or gambia or "georgia (republic)" or georgian or ghana or gold coast or gibraltar or greece or grenada or guam or guatemala or guinea or guinea bissau or guyana or british guiana or haiti or hispaniola or honduras or hungary or india or indonesia or timor or iran or iraq or isle of man or jamaica or jordan or kazakhstan or kazakh or kenya or "democratic people’s republic of korea" or republic of korea or north korea or south korea or korea or kosovo or kyrgyzstan or kirghizia or kirgizstan or kyrgyz republic or kirghiz or laos or lao pdr or "lao people's democratic republic" or latvia or lebanon or lebanese republic or lesotho or basutoland or liberia or libya or libyan arab jamahiriya or lithuania or macau or macao or republic of north macedonia or macedonia or madagascar or malagasy republic or malawi or nyasaland or malaysia or malay federation or malaya federation or maldives or indian ocean islands or indian ocean or mali or malta or micronesia or federated states of micronesia or kiribati or marshall islands or nauru or northern mariana islands or palau or tuvalu or mauritania or mauritius or mexico or moldova or moldovian or mongolia or montenegro or morocco or ifni or mozambique or portuguese east africa or myanmar or burma or namibia or nepal or netherlands antilles or nicaragua or niger or nigeria or oman or muscat or pakistan or panama or papua new guinea or new guinea or paraguay or peru or philippines or philipines or phillipines or phillippines or poland or "polish people's republic" or portugal or portuguese republic or puerto rico or romania or russia or russian federation or ussr or soviet union or union of soviet socialist republics or rwanda or ruanda or samoa or pacific islands or polynesia or samoan islands or navigator island or navigator islands or "sao tome and principe" or saudi arabia or senegal or serbia or seychelles or sierra leone or slovakia or slovak republic or slovenia or melanesia or solomon island or solomon islands or norfolk island or norfolk islands or somalia or south africa or south sudan or sri lanka or ceylon or "saint kitts and nevis" or "st. kitts and nevis" or saint lucia or "st. lucia" or "saint vincent and the grenadines" or saint vincent or "st. vincent" or grenadines or sudan or suriname or surinam or dutch guiana or netherlands guiana or syria or syrian arab republic or tajikistan or tadjikistan or tadzhikistan or tadzhik or tanzania or tanganyika or thailand or siam or timor leste or east timor or togo or togolese republic or tonga or "trinidad and tobago" or trinidad or tobago or tunisia or turkey or turkmenistan or turkmen or uganda or ukraine or uruguay or uzbekistan or uzbek or vanuatu or new hebrides or venezuela or vietnam or viet nam or middle east or west bank or gaza or palestine or yemen or yugoslavia or zambia or zimbabwe or northern rhodesia or global south or africa south of the sahara or sub-saharan africa or subsaharan africa or africa, central or central africa or africa, northern or north africa or northern africa or magreb or maghrib or sahara or africa, southern or southern africa or africa, eastern or east africa or eastern africa or africa, western or west africa or western africa or west indies or indian ocean islands or caribbean or central america or latin america or "south and central america" or south america or asia, central or central asia or asia, northern or north asia or northern asia or asia, southeastern or southeastern asia or south eastern asia or southeast asia or south east asia or asia, western or western asia or europe, eastern or east europe or eastern europe or developing country or developing countries or developing nation? or developing population? or developing world or less developed countr* or less developed nation? or less developed population? or less developed world or lesser developed countr* or lesser developed nation? or lesser developed population? or lesser developed world or under developed countr* or under developed nation? or under developed population? or under developed world or underdeveloped countr* or underdeveloped nation? or underdeveloped population? or underdeveloped world or middle income countr* or middle income nation? or middle income population? or low income countr* or low income nation? or low income population? or lower income countr* or lower income nation? or lower income population? or underserved countr* or underserved nation? or underserved population? or underserved world or under served countr* or under served nation? or under served population? or under served world or deprived countr* or deprived nation? or deprived population? or deprived world or poor countr* or poor nation? or poor population? or poor world or poorer countr* or poorer nation? or poorer population? or poorer world or developing econom* or less developed econom* or lesser developed econom* or under developed econom* or underdeveloped econom* or middle income econom* or low income econom* or lower income econom* or low gdp or low gnp or low gross domestic or low gross national or lower gdp or lower gnp or lower gross domestic or lower gross national or lmic or lmics or third world or lami countr* or transitional countr* or emerging economies or emerging nation?).ti,ab,jn,kf. (1637941)  2 (afghan or afghans or afghani or albanian? algerian? or american samoan? or angolan? or antiguan? or barbudan? or argentine? or argentinian? or argentinean? or armenian? or aruban? or azerbaijani? or bahraini? or bangladeshi? or bangalees or bajan? or belarusian? or byelorussian? or belizean? or beninese? or bhutanese or bolivian? or bosnian? or botswana or batswana or brazilian? or brasilian? or bulgarian? or burkinabe or burkinese or burundian? or cape verdean? or cabo verdean? or cambodian? or khmer or cameroonian? or central african? or chadian? or chilean? or chinese or colombian? or comorian? or congolese or costa rican? or ivorian? or croatian? or cuban? or cypriot? or czech? or djiboutian? or dominican? or ecuadorian? or egyptian? or salvadoran? or equatorial guinean? or equatoguinean? or eritrean? or estonian? or swazi? or swati? or ethiopian? or fijian or gabonese or gabonaise or gambian? or georgian? or ghanaian? or gibraltarian? or greek? or grenadian? or guamanian? or guatemalan? or guinean? or bissau guinean? or guyanese or haitian? or honduran? or hungarian? or indian? or indonesian? or iranian? or iraqian? or iraqi? or manx or jamaican? or jordanian? or kazakhstani? or kenyan? or kirabati or kirabatian? or north korean? or korean? or kosovar? or kosovan? or kyrgyz* or lao or laotian? or latvian? or lebanese or lesothan? or lesothonian? or mosotho or basotho or liberian? or libyan? or lithuanian? or macanese or macedonian? or malagasy or madagascan? or malawian? or malaysian? or maldivian? or malian? or maltese or marshallese? or mauritanian? or mauritian? or mexican? or micronesian? or moldovan? or mongolian? or mongol or montenegrin? or moroccan? or mozambican? or burmese or myanma or namibian? or nauruan? or nepali or nepalese or netherlands antillean? or nicaraguan? or nigerien? or nigerian? or northern mariana islander? or mariana? or omani? or pakistani? or palauan? or panamanian? or papua new guinean? or paraguayan? or peruvian? or philippine? or philipine? or phillipine? or phillippine? or filipino? or filipina? or polish or pole or poles or portuguese or puerto rican? or romanian? or russian? or soviet people or soviet population or rwandan? or rwandese or ruandan? or ruandese or samoan? or sao tomean? or santomean? or saudi arabian? or saudi? or senegalese or serbian? or montenegrin? or seychellois or seychelloise? or sierra leonean? or slovak? or slovene? or solomon islander? or somali? or south african? or south sudanese or sri lankan? or ceylonese or kittitian? or nevisian? or saint lucian? or vincentian? or sudanese or surinamese? or syrian? or tajik? or tajikistani? or tanzanian? or tanganyikan? or thai or timorese? or togolese or tongan? or trinidadian? or tobagonian? or tunisian? or turk? or turkish or turkmen? or tuvaluan? or ugandan? or ukrainian? or uruguayan? or uzbek? or vanuatu* or venezuelan? or vietnamese or yemeni? or yemenite? or yemenese or yugoslav? or yugoslavian? or zambian? or zimbabwean? or african? or asian? or pacific islander? or latin american? or central american? or south american? or caribbean? or west indian? or iberoamerican? or middle eastern?).ti,ab,jn,kf. (1203490)  3 Refugees/ (12357)  4 (Refugee* or migrant*).ti,ab,kw,kf. (34767)  5 1 or 2 or 3 or 4 (2407985) | 3ie LMIC cluster (with additional terms for refugees per review inclusion criteria (for refugee camps) |
| 6 exp Disasters/ (95896)  7 Emergencies/ (42570)  8 exp Relief Work/ (5830)  9 CRISIS INTERVENTION/ (6054)  10 Humanitarian.af. (7696)  11 (disaster* or emergenc* or "mass* casualt*" or catastrophe* or calamit* or cataclysmic or crisis or crises or outbreak* or "out break*").ti,ab,kw,kf. (672562)  12 Earthquakes/ (4536)  13 Landslides/ (221)  14 Volcanic Eruptions/ (1173)  15 Avalanches/ (191)  16 (Geophysical or earthquake* or aftershock* or foreshock* or volcano* or volcanic or lava or landslide* or "land slide*" or mudslide* or mudflow* or avalanche* or sinkhole* or "sink hole*").ti,ab,kw,kf. (24648)  17 Floods/ (3523)  18 Tidal Waves/ (377)  19 Tsunamis/ (1008)  20 (Hydrological or strom* or flood* or tsunami* or tidal wave* or "limnic erupt*" or thunderstorm*).ti,ab,kw,kf. (168822)  21 exp Climate Change/ (26575)  22 Droughts/ (10752)  23 Wildfires/ (899)  24 Extreme Weather/ (94)  25 (Climatological or climate or climatic or (global* adj2 warm*) or "heat wave*" or drought* or famine* or fire* or bushfire* or wildfire* or ((extrem* or harsh or severe*) adj3 (weather or temperature* or heat or hot or cold or summer* or winter*))).ti,ab,kw,kf. (217039)  26 Cyclonic Storms/ (2744)  27 (Meteorological or weather* or storm* or blizzard* or cyclone* or typhoon* or hurricane* or tornado* or dust storm* or duststrom*).ti,ab,kw,kf. (67744)  28 (Biologic* or epidemic* or pandemic* or plague* or infestation* or infectious disease*).ti,ab,kw,kf. (1397227)  29 Armed Conflicts/ (1151)  30 (war* or genocide* or genocidal* or conflict* or violence or violent* or invasion* or uprising or coup* or military or militant* or terrorist* or "arab* spring" or ((politic* or election* or government* or election*) adj3 (disturbance* or cris* or protest* or disput* or collaps* deadlock)) or riot* or displacement* or displaced* or evacuat* or ((organised or arm*) adj2 violen*)).ti,ab,kw,kf. (1718801)  31 ((aeroplane or plane or jet or train*) adj3 (crash* or derail* or fire)).ti,ab,kw,kf. (689)  32 Air Pollution/ (36873)  33 ((industrial or environment* or air or (human adj3 made) or (man adj3 made)) adj2 (accident* or degradation or pollution or destruction or hazard*)).ti,ab,kw,kf. (58681)  34 ((economic* or currenc*) adj3 (crises or instability or unstable or collapse)).ti,ab,kw,kf. (1009)  35 6 or 7 or 8 or 9 or 10 or 11 or 12 or 13 or 14 or 15 or 16 or 17 or 18 or 19 or 20 or 21 or 22 or 23 or 24 or 25 or 26 or 27 or 28 or 29 or 30 or 31 or 32 or 33 or 34 (3957221) | Humanitarian (settings cluster) |
| 36 ((early or rapid*) adj4 (warning or warned or alert* or alarm* or action* or respons* or monitor or monitoring or model or models or forecast* or "fore cast*" or notification* or notify)).ti,ab,kw,kf. (109787)  37 (warning system or warning scoring).ti,ab,kw,kf. (2508)  38 exp *Economics/ (344304)  39 (finance or financial or microfinance or micro finance or economic* or cash* or bank* or borrowing or borrow or loan or loans or cost* or income* or capital or buy or buying or sell or selling or poverty or destitute or destitution or saving or savings or credit or insurance or insurable or insured or "risk shar*" or "risk transf*" or premium or overdraft or "over draft" or asset or assets or wage or income or salary or (social adj3 (support or safety))).ti,ab,kw,kf. (1447869)  40 (cash or cashless or voucher* or ration* or "in-kind" or transfer or gift* or cheque* or economy or "non-food" or stamp or stamps or barter or exchange or credit*).ti,ab,kw,kf. (1050688)  41 exp Agriculture/ (115578)  42 Livestock/ (4941)  43 (Agriculture or (Agri adj1 culture) or livestock or animal or animals or crop* or seeds or farm* or (food adj2 produc*) or technology or infrastructure or feed or grain or grazing).ti,ab,kw,kf. (2007154)  44 Nutrition Therapy/ or exp Malnutrition/ (134072)  45 (nutrient* or nutrition* or nutritious or diet* or food* or feeding or supplement* or fortif* or vitamin* or healthy).ti,ab,kw,kf. (2756551)  46 (market* adj3 (based or recover* or connect* or infrastructure or function*)).ti,ab,kf,kw. (3333)  47 *water/ (67093)  48 drinking water/ (10399)  49 exp Water Supply/ (34742)  50 *Sanitation/ (4960)  51 *Water Purification/ (28238)  52 (Water or aqua or hydration or sanitation* or rainwater*).ti,ab,kw,kf. (933876)  53 36 or 37 or 38 or 39 or 40 or 41 or 42 or 43 or 44 or 45 or 46 or 47 or 48 or 49 or 50 or 51 or 52 (7312984) | This is the ‘interventions’ cluster, based on the work in the protocol. |
| 54 35 or 53 (10102020) | This line combines humanitarian terms OR interventions terms. |
| 55 exp Food Security/ (431)  56 exp Food Supply/ (16013)  57 Food Assistance/ (1636)  58 *Food/ (20090)  59 (Food* or feed* or nutrition* or calorie*).ti,ab,kw,kf. (1254757)  60 55 or 56 or 57 or 58 or 59 (1261384) | Broad Food – broad because line 59 is not adjacent to terms for food security. |
| 61 Program Evaluation/ or evaluation study/ (320200)  62 ((match* adj2 (propensity or coarsened or covariate or neighbo?r)) or "propensity score" or ("difference* in difference*" or "difference-in-difference*" or "differences-in-difference*" or "double difference*") or (quasi-experiment$2 or "quasi experiment$2") or (estimator and evaluat*) or ("instrumental variable*" or (IV adj2 (estimation or approach))) or (Heckman adj3 (model* or approach*)) or ((two-stage or "two stage") adj3 (control* or function* or "least squares")) or "regression discontinuity" or "time series" or counterfactual or "segment* regression" or (non adj2 participant*) or ((control or comparison) adj2 (group* or condition* or area* or village* or household* or intervention)) or (panel$1 adj2 (data or household* or model*)) or ((exploit* or "tak* advantage") adj3 (variation* or variety or exogen* or heterogen*)) or (econometric adj2 (model* or adjust*)) or (select* adj2 (bias* or self))).ti,ab,kw,kf. (773816)  63 ((experiment$4 adj2 (design or study or research or evaluation or evidence or vary or varies or variation)) or ((random or randomi#ed or randomly) adj2 (trial or assign* or treatment or control* or allocat* or experiment$2 or vary or varies or variation or choose or chose*))).ti,ab,kw,kf. (811427)  64 ("program* evaluation" or "project evaluation" or "evaluation research" or "natural experiment*" or "program* effectiveness" or "outcome assessment" or "evaluation study" or "field experiment").ti,ab,kw,kf. (33594)  65 ((impact? or effect*) adj2 (evaluat* or assess or assessing or assessment or analyze or analyse or analyzing or analysing or analysis or analytical or estimate or estimating or estimation or cause or causal)).ti,ab,kw,kf. (544836)  66 "Systematic Review"/ (200284)  67 ((Systematic* or synthes*) adj3 (research or evaluation* or overview or finding* or thematic* or report or descriptive or explanatory or narrative or meta* or review* or data or literature or studies or evidence or map or mapping or quantitative or study or studies or paper or impact or impacts or effect* or compar*)).ti,ab,kw,kf. (472928)  68 ("Meta regression" or "meta synth*" or "meta-synth*" or "meta analy*" or "metaanaly*" or "meta-analy*" or "metanaly*" or "Metaregression" or "Meta-regression" or "Methodologic* overview" or "pool* analys*" or "pool* data" or "Quantitative* overview" or "research integration").ti,ab,kw,kf. (258081)  69 ((effectiveness or effects or systemat* or synth* or integrat* or gap or methodologic* or quantitative or evidence or literature or rapid or scoping) adj3 (review or map)).ti,ab,kw,kf. (576473)  70 61 or 62 or 63 or 64 or 65 or 66 or 67 or 68 or 69 (2932143) | 3ie search filters for IE and SR |
| 71 (2000* or 2001* or 2002* or 2003* or 2004* or 2005* or 2006* or 2007* or 2008* or 2009* or 2010* or 2011* or 2012* or 2013* or 2014* or 2015* or 2016* or 2017* or 2018* or 2019* or 2020* or 2021* or 2022*).dt,dp,ed,ep,yr. (22935560)  72 5 and 54 and 60 and 70 and 71 (20994) | Line 72 combines the search.  5 = LMIC terms  54 = humanitarian OR intervention terms  60 = broad food  71 = 3ie methods terms |

These three studies are blocked by the LMIC cluster

1.

Evidence on Child Nutrition Recommendations and Challenges in Crisis Settings: A Scoping Review. [Review]

Marshall AI, Lasco G, Phaiyarom M, Pangkariya N, Leuangvilay P, Sinam P, Suphanchaimat R, Julchoo S, Kunpeuk W, Zhang Y

International Journal of Environmental Research & Public Health [Electronic Resource]. 18(12), 2021 06 20.

[Journal Article. Research Support, Non-U.S. Gov't. Review]

UI: 34203109

Adequate child nutrition is critical to child development, yet child malnutrition is prevalent in crisis settings. However, the intersection of malnutrition and disasters is sparse. This study reviews existing evidence on nutrition responses and outcomes for infants and young children during times of crisis. The scoping review was conducted via two approaches: a systematic search and a purposive search. For the systematic search, two key online databases, PubMed and Science Direct, were utilized. In total, data from 32 studies were extracted and included in the data extraction form. Additionally, seven guidelines and policy documents were included, based on relevance to this study. Overall, the existing evidence demonstrates the negative impacts of crises on nutritional status, diet intake, anthropometric failure, and long-term child development. On the other hand, crisis-related interventions positively affected nutrition-related knowledge and practices. Further studies should be carried out to explore the sustainability of the interventions and the success of existing guidelines. Since this study focuses only on nutrition among children under three, further studies should likewise consider an extended age range from three to five years.

Version ID

1

Status

MEDLINE

Author NameID

Marshall, Aniqa Islam; ORCID: <https://orcid.org/0000-0001-6575-731X> Lasco, Gideon; ORCID: <https://orcid.org/0000-0002-6402-682X>

Phaiyarom, Mathudara; ORCID: <https://orcid.org/0000-0002-1156-1984>

Pangkariya, Nattanicha; ORCID: <https://orcid.org/0000-0001-9393-6593>

Suphanchaimat, Rapeepong; ORCID: <https://orcid.org/0000-0002-3664-9050>

Authors Full Name

Marshall, Aniqa Islam, Lasco, Gideon, Phaiyarom, Mathudara, Pangkariya, Nattanicha, Leuangvilay, Phetdavanh, Sinam, Pigunkaew, Suphanchaimat, Rapeepong, Julchoo, Sataporn, Kunpeuk, Watinee, Zhang, Yunting

Institution

Marshall, Aniqa Islam. International Health Policy Program, Ministry of Public Health, Nonthaburi 1100, Thailand. Lasco, Gideon. Department of Anthropology, University of the Philippines Diliman, Quezon City 1107, Philippines.

Lasco, Gideon. Development Studies Program, Ateneo de Manila University, Diliman, Quezon City 1106, Philippines.

Lasco, Gideon. Equity Initiative, Bangkok 10110, Thailand.

Phaiyarom, Mathudara. International Health Policy Program, Ministry of Public Health, Nonthaburi 1100, Thailand.

Pangkariya, Nattanicha. International Health Policy Program, Ministry of Public Health, Nonthaburi 1100, Thailand.

Leuangvilay, Phetdavanh. Equity Initiative, Bangkok 10110, Thailand.

Sinam, Pigunkaew. International Health Policy Program, Ministry of Public Health, Nonthaburi 1100, Thailand.

Suphanchaimat, Rapeepong. International Health Policy Program, Ministry of Public Health, Nonthaburi 1100, Thailand.

Suphanchaimat, Rapeepong. Equity Initiative, Bangkok 10110, Thailand.

Suphanchaimat, Rapeepong. Division of Epidemiology, Department of Disease Control, Ministry of Public Health, Nonthaburi 11000, Thailand.

Julchoo, Sataporn. International Health Policy Program, Ministry of Public Health, Nonthaburi 1100, Thailand.

Kunpeuk, Watinee. International Health Policy Program, Ministry of Public Health, Nonthaburi 1100, Thailand.

Zhang, Yunting. Equity Initiative, Bangkok 10110, Thailand.

Zhang, Yunting. Child Health Advocacy Institute, Shanghai Children's Medical Center, Shanghai Jiao Tong University School of Medicine, Shanghai 200127, China.

Year of Publication

2021

3.

Comparative effectiveness of an economic empowerment program on adolescent economic assets, education and health in a humanitarian setting.

Glass N, Remy MM, Mayo-Wilson LJ, Kohli A, Sommer M, Turner R, Perrin N

BMC Public Health. 20(1):170, 2020 Feb 04.

[Comparative Study. Journal Article. Randomized Controlled Trial]

UI: 32019539

BACKGROUND: Adolescence is a critical period of human development, however, limited research on programs to improve health and well-being among younger adolescents living in conflict-affected and humanitarian settings exists. The purpose of this study was to assess the comparative effectiveness of an economic empowerment program on young adolescent outcomes in a complex humanitarian setting.

METHODS: This longitudinal, mixed methods study examined the relative effectiveness of an integrated parent (Pigs for Peace, PFP) and young adolescent (Rabbits for Resilience, RFR) animal microfinance/asset transfer program (RFR + PFP) on adolescent outcomes of asset building, school attendance, mental health, experienced stigma, and food security compared to RFR only and PFP only over 24 months. A sub-sample of young adolescents completed in-depth qualitative interviews on the benefits and challenges of participating in RFR.

RESULTS: Five hundred forty-two young adolescents (10-15 years) participated in three groups: RFR + PFP (N = 178), RFR only (N = 187), PFP only (N = 177). 501 (92.4%) completed baseline surveys, with 81.7% (n = 442) retention at endline. The group by time interaction (24 months) was significant for adolescent asset building (X2 = 16.54, p = .002), school attendance (X2 = 12.33, p = .015), and prosocial behavior (X2 = 10.56, p = .032). RFR + PFP (ES = 0.31, ES = 0.38) and RFR only (ES-0.39, ES = 0.14) adolescents had greater improvement in asset building and prosocial behavior compared to PFP only, respectively. The odds of missing two or more days of school in the past month were 78.4% lower in RFR only and 45.1% lower in RFR + PFP compared to PFP only. No differences between groups in change over time were found for internalizing behaviors, experienced stigma, or food security. Differences by age and gender were observed in asset building, prosocial behavior, school attendance, experienced stigma, and food security. The voices of young adolescents identified the benefits of the RFR program through their ability to pay for school fees, help their families meet basic needs, and the respect they gained from family and community. Challenges included death of rabbits and potential conflict within the household on how to use the rabbit asset.

CONCLUSION: These findings underscore the potential for integrating economic empowerment programs with both parents and young adolescents to improve economic, educational, and health outcomes for young adolescents growing up in rural and complex humanitarian settings.

TRIAL REGISTRATION: NCT02008695. Retrospectively registered 11 December 2013.

Version ID

1

Status

MEDLINE

Author NameID

Glass, Nancy; ORCID: <http://orcid.org/0000-0002-6691-3684>

Authors Full Name

Glass, Nancy, Remy, Mitima Mpanano, Mayo-Wilson, Larissa Jennings, Kohli, Anjalee, Sommer, Marni, Turner, Rachael, Perrin, Nancy

Institution

Glass, Nancy. School of Nursing, Johns Hopkins University, Baltimore, Maryland, USA. nglass1@jhu.edu. Remy, Mitima Mpanano. Programme d'Appui aux Initiatives Economiques (PAIDEK), Bukavu, Democratic Republic of Congo.

Mayo-Wilson, Larissa Jennings. School of Public Health, Johns Hopkins University, Baltimore, Maryland, USA.

Mayo-Wilson, Larissa Jennings. School of Public Health, Indiana University, Bloomington, Indiana, USA.

Kohli, Anjalee. Institute of Reproductive Health, Georgetown University, Washington, District of Columbia, USA.

Sommer, Marni. Mailman School of Public Health, Columbia University, New York, New York, USA.

Turner, Rachael. School of Nursing, Johns Hopkins University, Baltimore, Maryland, USA.

Perrin, Nancy. School of Nursing, Johns Hopkins University, Baltimore, Maryland, USA.

Year of Publication

2020

5.

The Role of SNAP in Home Food Availability and Dietary Intake among WIC Participants Facing Unstable Housing.

Bruening M, McClain D, Moramarco M, Reifsnider E

Public Health Nursing. 34(3):219-228, 2017 05.

[Journal Article]

UI: 28084013

OBJECTIVE: Little nutrition research has been conducted among families with unstable housing. The objective of this study was to examine the role of food stamps (i.e., Supplemental Nutrition Assistance Program; SNAP) in home food availability and dietary intake among WIC families who experienced unstable housing.

DESIGN AND SAMPLE: Cross-sectional study among vulnerable families. Low-income, multiethnic families with children participating in WIC (n = 54).

MEASURES: Dietary intake was assessed with 24-hr recalls. Home food availability was assessed with an adapted home food inventory for low-income, multiethnic families. Validation results from adapted home food inventory for these families are also reported.

RESULTS: SNAP households had more foods than non-SNAP households; few significant associations were observed between food availability and child dietary intake.

CONCLUSIONS: With few exceptions, the home food environment was not related to children's dietary intake among these vulnerable families. More research is needed on food access for families facing unstable housing.

Copyright © 2017 Wiley Periodicals, Inc.

Version ID

1

Status

MEDLINE

Authors Full Name

Bruening, Meg, McClain, Darya, Moramarco, Michael, Reifsnider, Elizabeth

Institution

Bruening, Meg. School of Nutrition and Health Promotion, Arizona State University, Phoenix, Arizona. McClain, Darya. College of Nursing and Health Innovation, Arizona State University, Phoenix, Arizona.

Moramarco, Michael. College of Nursing and Health Innovation, Arizona State University, Phoenix, Arizona.

Reifsnider, Elizabeth. College of Nursing and Health Innovation, Arizona State University, Phoenix, Arizona.

Year of Publication

2017

--

These two studies are blocked by the broad food cluster

2.

Delivering water, sanitation and hygiene interventions to women and children in conflict settings: a systematic review.

Als D, Meteke S, Stefopulos M, Gaffey MF, Kamali M, Munyuzangabo M, Shah S, Jain RP, Radhakrishnan A, Siddiqui FJ, Ataullahjan A, Bhutta ZA

BMJ Global Health. 5(Suppl 1), 2020 07.

[Journal Article. Research Support, Non-U.S. Gov't. Systematic Review]

UI: 32641288

BACKGROUND: Access to safe water and sanitation facilities and the adoption of effective hygiene practices are fundamental to reducing maternal and child morbidity and mortality globally. In armed conflict settings, inadequate water, sanitation and hygiene (WASH) infrastructure poses major health risks for women and children. This review aimed to synthesise the existing information on WASH interventions being delivered to women and children in conflict settings in low-income and middle-income countries (LMICs) and to identify the personnel, sites and platforms being used to deliver such interventions.

METHODS: We conducted a systematic search for publications indexed in four databases, and grey literature was searched through the websites of humanitarian agencies and organisations. Eligible publications reported WASH interventions delivered to conflict-affected women or children. We extracted and synthesised information on intervention delivery characteristics, as well as barriers and facilitators.

RESULTS: We identified 58 eligible publications reporting on the delivery of WASH interventions, mostly in Sub-Saharan Africa. Non-Governmental Organization (NGO)/United Nations (UN) agency staff were reported to be involved in delivering interventions in 62% of publications, with the most commonly reported delivery site being community spaces (50%). Only one publication reported quantitative data on intervention effectiveness among women or children.

DISCUSSION: This review revealed gaps in the current evidence on WASH intervention delivery in conflict settings. Little information is available on the delivery of water treatment or environmental hygiene interventions, or about the sites and personnel used to deliver WASH interventions. Limited quantitative data on WASH intervention coverage or effectiveness with respect to women or children are important gaps, as multiple factors can affect how WASH services are accessed differently by women and men, and the hygiene needs of adolescent girls and boys differ; these factors must be taken into account when delivering interventions in conflict settings.

PROSPERO REGISTRATION NUMBER: CRD42019125221.

Copyright © Author(s) (or their employer(s)) 2020. Re-use permitted under CC BY. Published by BMJ.

Version ID

1

Status

MEDLINE

Author NameID

Shah, Shailja; ORCID: <https://orcid.org/0000-0003-2329-5027> Jain, Reena P; ORCID: <https://orcid.org/0000-0001-7004-5093>

Authors Full Name

Als, Daina, Meteke, Sarah, Stefopulos, Marianne, Gaffey, Michelle F, Kamali, Mahdis, Munyuzangabo, Mariella, Shah, Shailja, Jain, Reena P, Radhakrishnan, Amruta, Siddiqui, Fahad J, Ataullahjan, Anushka, Bhutta, Zulfiqar A

Institution

Als, Daina. Centre for Global Child Health, Hospital for Sick Children, Toronto, Ontario, Canada. Meteke, Sarah. Centre for Global Child Health, Hospital for Sick Children, Toronto, Ontario, Canada.

Stefopulos, Marianne. Centre for Global Child Health, Hospital for Sick Children, Toronto, Ontario, Canada.

Gaffey, Michelle F. Centre for Global Child Health, Hospital for Sick Children, Toronto, Ontario, Canada.

Kamali, Mahdis. Centre for Global Child Health, Hospital for Sick Children, Toronto, Ontario, Canada.

Munyuzangabo, Mariella. Centre for Global Child Health, Hospital for Sick Children, Toronto, Ontario, Canada.

Shah, Shailja. Centre for Global Child Health, Hospital for Sick Children, Toronto, Ontario, Canada.

Jain, Reena P. Centre for Global Child Health, Hospital for Sick Children, Toronto, Ontario, Canada.

Radhakrishnan, Amruta. Centre for Global Child Health, Hospital for Sick Children, Toronto, Ontario, Canada.

Siddiqui, Fahad J. Centre for Global Child Health, Hospital for Sick Children, Toronto, Ontario, Canada.

Siddiqui, Fahad J. Health Services and Systems Research, Duke-NUS Graduate Medical School, Singapore.

Ataullahjan, Anushka. Centre for Global Child Health, Hospital for Sick Children, Toronto, Ontario, Canada.

Bhutta, Zulfiqar A. Centre for Global Child Health, Hospital for Sick Children, Toronto, Ontario, Canada zulfiqar.bhutta@sickkids.ca.

Bhutta, Zulfiqar A. Center of Excellence in Women and Child Health, Aga Khan University, Karachi, Pakistan.

Year of Publication

2020

4.

Does 'Manna from Heaven' help? The role of cash transfers in disaster recovery-lessons from Fiji after Tropical Cyclone Winston.

Ivaschenko O, Doyle J, Kim J, Sibley J, Majoka Z

Disasters. 44(3):455-476, 2020 Jul.

[Journal Article]

UI: 31583740

This paper contributes to the evidence on the effectiveness of shock-responsive social protection systems in helping affected households recover from the negative consequences of disasters. It evaluates the influence of the top-up cash transfers provided by the Government of Fiji to poor households in the wake of Tropical Cyclone Winston, which struck the Pacific Island country on 20 February 2016. The impact evaluation strategy incorporates a sharp regression discontinuity design to define treatment and control groups, based on the eligibility threshold of the poverty benefit scheme. The results indicate that treatment households-that is, those that received cash transfers-are significantly more likely to report quicker recovery from various shocks. Female-headed households are more likely to recover from the ramifications, whereas households with older heads are less likely to do so. The presence of a functioning market appears to be a major factor aiding the speed of recovery. Finally, the evidence points towards strong district effects on recovery.

Copyright © 2019 The Authors Disasters © 2019 Overseas Development Institute.

Version ID

1

Status

MEDLINE

Authors Full Name

Ivaschenko, Oleksiy, Doyle, Jesse, Kim, Jaekyun, Sibley, Jonathan, Majoka, Zaineb

Institution

Ivaschenko, Oleksiy. Senior Economist at the World Bank, United States. Doyle, Jesse. Economist at the World Bank, United States.

Kim, Jaekyun. Consultant at the World Bank, United States.

Sibley, Jonathan. Consultant at the World Bank, United States.

Majoka, Zaineb. Consultant at the World Bank, United States.

Year of Publication

2020
